# Climate-Driven Changes in the Habitat Suitability and Potential Co-Occurrence of Daurian Ground Squirrels and Pikas in Inner Mongolia: Implications for Brucellosis Surveillance

**DOI:** 10.3390/ani16213387

**Published:** 2026-10-27

**Authors:** Haiyan Jiang, Nan Yang, Jinyang Dong, Liyan Zhang, Liqiong Yan, Xiaoli Wang

**Affiliations:** College of Forestry, Inner Mongolia Agricultural University, Hohhot 010011, China

**Keywords:** *Spermophilus dauricus*, *Ochotona dauurica*, brucellosis, species distribution model, MaxEnt, climate change, joint co-occurrence index, path analysis, exploratory transmission-threshold modeling, next-generation matrix, Inner Mongolia

## Abstract

Where a multi-host pathogen can circulate depends on where its hosts overlap, not on any single host’s range, yet climate-change projections for zoonoses rarely model two hosts jointly or test how a warming signal is channeled toward their overlap. We addressed this gap for brucellosis, which remains endemic across the pastoral landscapes of Inner Mongolia, China, by building ENMeval-optimized MaxEnt niche models for two candidate sylvatic hosts (infection of which with *Brucella* in this region has not been established), the Daurian ground squirrel (*Spermophilus dauricus*, *n* = 620) and the Daurian pika (*Ochotona dauurica*, *n* = 17, with a three-method variable-screening pipeline and small-sample controls), projecting both onto current climate and eight CMIP6 scenarios (SSP1-2.6/2-4.5/3-7.0/5-8.5 × 2050s/2070s; validation AUC = 0.808 for the squirrel and 0.940 for the pika), combining them into a joint host co-occurrence index (JCI) that describes potential habitat overlap rather than disease risk, and fitting a path model that describes how climate anomaly relates to each host’s suitability and to the JCI. The two hosts diverge sharply: by the 2070s, suitable habitat contracts by 9.6–84.3% for the generalist ground squirrel but by >99.9% for the pika under the fitted model, whose projected collapse follows from a threshold in mean wettest-quarter temperature (≈18–19 °C) that is exceeded almost everywhere in the future; this loss is robust to occurrence filtering, regularization, binary thresholds and a second climate model but depends on the temperature variable and on climates beyond those sampled by the 17 records. This asymmetry carries over to the overlap: the current 66,737 km^2^ zone where both hosts co-occur shrinks by >99.9% under six of eight future scenarios, a collapse that is robust to how the joint index is constructed (geometric mean, minimum, or arithmetic mean all show the same pattern). The path model shows that climate anomaly is the better linear predictor of the squirrel’s own habitat trajectory (standardized β = −0.195 vs. −0.059 for the pika), whereas the pika’s habitat change has nearly double the standardized association with JCI change (β = 0.635 vs. 0.324); the latter association is largely a mathematical consequence of how the JCI is constructed, and under spatially corrected estimation, the climate effect on pika suitability is statistically indistinguishable from zero (95% CI −0.305 to 0.184). An exploratory next-generation-matrix calculation with uncalibrated parameters shows the same direction of change under most parameter combinations (the area with *R*_0_ > 1 declines under all but one) but is sensitive to its assumptions and is not a validated epidemiological prediction. These results identify the pika as the more informative indicator of change in host overlap for ecological monitoring; whether it matters more for brucellosis maintenance or spillover is unknown and requires serological, abundance and contact-rate data. The approach offers a reproducible template (joint niche modeling coupled to path analysis and exploratory transmission-threshold modeling) for exploring multi-host co-occurrence under climate change beyond this system.

## Simple Summary

Brucellosis is a bacterial disease shared by livestock, wildlife and people, and it remains a persistent public-health problem on the grasslands of Inner Mongolia, China. Two small burrowing mammals, the Daurian ground squirrel and the Daurian pika, are candidate sylvatic hosts that could maintain the bacterium in the landscape and bring it into contact with grazing livestock, although infection of these two species with *Brucella* has not been confirmed in this region. We used field survey and museum/citizen-science occurrence records together with climate, terrain and land-cover data to model where each species finds suitable habitat today, which environmental factors most shape that habitat, and how it is expected to change under future climate warming. We then combined the two species’ suitability maps into a simple joint index that highlights where both candidate hosts are likely to overlap on the landscape, as an ecological indicator of potential host overlap (not a measure of disease risk, because no disease case data were available), and used a path model to describe whether climate warming’s effect on this overlap index works mainly through the ground squirrel, through the pika, or both. Both species are projected to lose considerable suitable habitat by mid- and late-century, with the pika’s habitat contracting far more severely than the ground squirrel’s; consequently, the area where both hosts are jointly likely to occur nearly disappears under most future scenarios, and the path model indicates that changes in the pika’s habitat are more strongly associated with this decline in overlap, although part of this association follows from how the index is calculated, and the pika projection rests on only 17 occurrence records. These maps are a first step for ecological monitoring; deciding where disease surveillance should be prioritized will require data on infection, abundance and contact with livestock.

## 1. Introduction

Every year, tens of thousands of people across northern China contract brucellosis from the animals they herd. The disease can become chronic and damage joints and organs for years after the initial infection. Inner Mongolia Autonomous Region, whose grazing economy is among the largest in the country, has driven a resurgence in national incidence over the past two decades despite sustained livestock vaccination and culling programs [1,2,3]. This persistence suggests that control aimed only at livestock cannot eliminate a pathogen that also circulates among wild mammals because sylvatic reservoirs can maintain transmission chains that livestock-only interventions do not reach [4,5,6]. Overlooking wildlife leaves the underlying spatial risk unaddressed, as shown for another rodent-borne zoonosis, plague, where long-term environmental change has measurably shifted disease risk [7,8]. The more specific question is therefore which wild species act as reservoirs, and where on the steppe they are most likely to bring the pathogen into contact with livestock and with each other.

Two small burrowing mammals are repeatedly named as candidates: the Daurian ground squirrel (*Spermophilus dauricus*) and the Daurian pika (*Ochotona dauurica*) [9]. Both form dense, semi-fossorial colonies that put them in frequent contact with grazing livestock and, where their ranges overlap, with each other, a contact pattern epidemiologically relevant for a multi-host pathogen like *Brucella* [10,11]. *S. dauricus* is known to carry other zoonotic agents (*Anaplasma*, *Bartonella* and *Rickettsia*) in the wild [12], which shows that it can host zoonotic pathogens; however, we are not aware of published serological or isolation data showing *Brucella* infection in either species in Inner Mongolia, so their status as brucellosis hosts is a hypothesis that this study does not test. The two hosts differ, however, in their climatic tolerance. The ground squirrel is a broadly distributed grassland generalist, tolerant of the steppe’s full seasonal temperature swing [13,14]. Pikas, across the genus *Ochotona*, are the opposite: thermally constrained specialists that already show measurable range contraction under warming elsewhere. The American pika (*O. princeps*) has undergone documented local extirpations tied to summer heat exposure [15,16,17], and a landscape-scale resurvey of small-mammal communities in Yosemite found the same divergence that we test here: heat-tolerant, generalist rodents expanding upslope while thermally sensitive specialists contracted [18]. If that asymmetry holds for these two hosts, then climate change will not shrink their ranges in parallel; it will reshape the geography of where they overlap, and, by extension, of the opportunities for cross-species contact that their co-occurrence could create, in ways no single-species forecast can show.

Species distribution models (SDMs) are the standard tool for making that kind of forecast: given occurrence records and environmental layers, they characterize a species’ realized niche and project it onto future climate surfaces [19,20], and tuning frameworks such as ENMeval have made it routine to fit a single species’ Maximum Entropy (MaxEnt) model rigorously rather than on default settings [21,22,23,24,25,26]. They are not designed, however, to address the question posed by a multi-host system. Where a multi-host pathogen can circulate is not the sum of its hosts’ individual ranges; it is set by where those ranges converge, and that convergence can be disproportionately sensitive to whichever host’s climate response is the most fragile, not the most typical, which is exactly the asymmetry suggested by the ecological contrast above. This raises two questions that a single-species SDM cannot answer: how do two independently modeled hosts’ suitability surfaces combine into a JCI that neither model specifies on its own, and once that joint surface is defined, is its response to climate change explained equally by both hosts, or does it simply inherit the fragility of whichever host is the weaker link? Answering the second question needs an explicit path- or mediation-analytic framework [27,28] applied downstream of the niche models themselves, a combination that, to our knowledge, has not previously been applied to joint host-distribution modeling for a zoonotic disease system.

Here we combine these approaches for the Inner Mongolia system. Using georeferenced occurrence records for both species within Inner Mongolia, we applied a three-method variable-screening pipeline, fitted ENMeval-optimized MaxEnt models for each species, and projected both onto current climate and eight CMIP6 future scenarios spanning two time horizons (2050s, 2070s) and four Shared Socioeconomic Pathways (SSP1-2.6, SSP2-4.5, SSP3-7.0, SSP5-8.5). We combined the two resulting suitability surfaces into a joint host co-occurrence index (JCI), an ecological indicator of potential host overlap that is not a measure of disease risk because georeferenced disease case data were unavailable and extended this static index into an exploratory epidemic-threshold calculation using a next-generation-matrix formulation of the basic reproduction number, treating the unknown cross-species transmission efficiency as a sensitivity parameter rather than an assumed constant. We also tested how robust the pika projections are to occurrence filtering, model specification, binary thresholds and a second climate model. Finally, we fitted a grid-cell-level path model linking climate anomaly, each species’ suitability change, and JCI change to formally describe the pathway implied by the ecological asymmetry between the two hosts. We hypothesized that the pika’s narrower climatic niche, not the ground squirrel’s much larger range, would prove to be more strongly associated with future change in dual-host overlap.

## 2. Materials and Methods

### 2.1. Study Area

The study was conducted across the Inner Mongolia Autonomous Region, China (approximately 97.17–126.08° E, 37.38–53.34° N), a region spanning temperate steppe, desert-steppe and forest-steppe biomes along a pronounced west-to-east precipitation gradient and comprising 12 prefecture-level divisions (9 prefecture-level cities and 3 leagues). The region’s extensive grazing economy and long history of endemic brucellosis make it a representative setting for evaluating potential wildlife-host co-occurrence, consistent with documented spatial heterogeneity in grazing pressure and rangeland condition across the broader Mongolian Plateau [29] (Figure 1).

### 2.2. Species Occurrence Data

Ground squirrel occurrence records (*n* = 620) were compiled from a systematic rodent field-survey dataset covering Inner Mongolia, retaining records unambiguously identified as *Spermophilus dauricus* and using the surveyed WGS84 coordinates directly. Pika occurrence data were sparser: 15 field-survey records were supplemented with occurrence data retrieved through the application programming interface (API) of the Global Biodiversity Information Facility (GBIF) [30] on 31 August 2026 for *Ochotona dauurica*, filtered to records with coordinates and excluding fossil specimens. Because the initial bounding-box filter used for the GBIF query (approximately 95–128° E, 35–55° N) extended well beyond Inner Mongolia into Mongolia and neighboring Chinese provinces, all candidate points (field survey and GBIF combined) were subsequently intersected with the study-area boundary; only records falling inside Inner Mongolia were retained for model fitting, background sampling and prediction since the environmental covariate layers are themselves restricted to this extent. This left 17 pika occurrence records (15 field-survey, 2 GBIF-derived) for model fitting, a markedly smaller sample than for the ground squirrel, which we handle by restricting MaxEnt feature-class complexity for the pika model and by interpreting pika-derived results with caution. Because the records were retrieved through the API rather than as a citable download, the two GBIF-derived records retained within Inner Mongolia are also cited at the dataset level: CAS Mammalogy (MAM; https://doi.org/10.15468/dhbozg) and the International Barcode of Life project (iBOL; https://doi.org/10.15468/inygc6) [30]. The records were not collected through a standardized survey of the species’ range, and no bias layer (for example, a target-group background) was available, so sampling bias cannot be excluded; the spatial clustering and climatic coverage of the pika records are quantified in Section 3.10.

### 2.3. Environmental Variables and Variable Selection

Twenty-six candidate predictors were assembled at ~4.6 km (0.0417°) resolution: 19 bioclimatic variables (bio1–bio19) following the WorldClim conventions [31,32]; elevation, slope and aspect derived from a digital elevation model; the global Human Footprint index [33]; distance to the nearest river; a normalized difference vegetation index (NDVI) composite; and a categorical land-cover classification. For future scenarios, the 19 bioclimatic layers were replaced with CMIP6-derived projections (BCC-CSM2-MR global circulation model, WorldClim 2.1-downscaled output at 2.5 arc-minute resolution) for the 2050s (2041–2060) and 2070s (2061–2080) under four Shared Socioeconomic Pathways (SSP1-2.6, SSP2-4.5, SSP3-7.0, SSP5-8.5) [34], while elevation, slope and aspect were held constant; land cover, human footprint, NDVI and river distance were held at a shared future baseline (identical across SSP/decade combinations, distinct from the current-day value) except where noted below.

The land-cover projection for the SSP3-7.0 scenario was incomplete in the source data: the 2070s layer was missing entirely, and the 2050s layer was found to be an exact duplicate of the SSP5-8.5 2050s land-cover raster (verified by checksum) rather than a genuine SSP3-7.0 projection. No alternative source for this layer was available. We therefore held land cover at its current-day value for the SSP3-7.0 scenario at both time horizons while still using the genuine SSP3-7.0 bioclimatic projections; this is a limitation of the study. All area, centroid and JCI statistics reported below were computed after masking the environmental raster stack and every derived suitability surface to the true Inner Mongolia administrative boundary since the source rasters are a rectangular clip that extends beyond the province’s irregular outline.

To reduce collinearity and dimensionality, we applied a three-method consensus variable-screening procedure separately for each species using 5000 randomly sampled background locations. First, pairwise Pearson correlations among all 26 candidates were computed, and variables were hierarchically clustered (complete linkage on 1 − |*r*|), cut at a height corresponding to |*r*| = 0.8 (Figure 2a). Second, stepwise variance inflation factor (VIF) elimination (threshold = 10) was applied using the usdm package [35] (Figure 2b). Third, permutation importance was estimated from a preliminary maxnet model (linear + quadratic features) fitted to all 26 candidates by measuring the drop in AUC after randomly permuting each variable. The final consensus set retained, within each correlation cluster, the VIF-surviving variable with the highest permutation importance. This procedure converged on an identical 12-variable set for both species: aspect, bio2 (mean diurnal temperature range), bio3 (isothermality), bio8 (mean temperature of wettest quarter), bio14 (precipitation of driest month), bio15 (precipitation seasonality), elevation, human footprint, land cover, NDVI, river distance and slope.

### 2.4. MaxEnt Model Development and Evaluation

MaxEnt models were fitted using the maxnet implementation [22] to avoid a Java dependency. For each species, 10,000 random background points were drawn from the 12-variable environmental stack. Model hyperparameters (feature class: linear [L], linear + quadratic [LQ], linear + quadratic + hinge [LQH], hinge-only [H]; regularization multiplier: 0.5–4.0) were tuned using ENMeval 2.0 [24], selecting the combination minimizing corrected Akaike Information Criterion (AICc) across candidate models. For the ground squirrel (*n* = 620), four-fold spatial block cross-validation was used. Given the pika’s very small sample (*n* = 17, below general recommended minima for reliable MaxEnt fitting [36]), we used leave-one-out (jackknife) cross-validation and restricted the tuning grid to the simpler L and LQ feature classes with regularization multiplier ≥ 1 since more flexible hinge-based feature classes produced models with more free coefficients than occurrence records and were judged too unstable to interpret [25]. Model discrimination was evaluated with the area under the receiver operating characteristic curve (AUC) on held-out partitions and with the continuous Boyce index where computable [37]. Variable importance for each species’ selected model was estimated by permutation (AUC drop after shuffling each predictor in turn) computed directly on the final model. A binary suitable/unsuitable threshold was defined at the 10th percentile of predicted suitability at training-presence locations for each species.

Given the pika’s small sample, we additionally tested whether its dominant predictor’s steep response threshold was a genuine signal or a single-point overfitting artifact by refitting the model 17 times (leave-one-out on occurrence points) and recording, on each refit’s response curve, the bio8 value at which predicted suitability crossed 0.5.

### 2.5. Current and Future Habitat Suitability Projections

Each species’ selected model was projected onto current climate and each of the eight future scenario combinations, producing continuous suitability surfaces (0–1) and binary suitable-habitat maps.

### 2.6. Interspecific Environmental Niche Overlap

The JCI asks whether the two species’ predicted geographic ranges overlap; it does not by itself say whether the two species occupy similar positions in environmental space, a distinct and complementary question. We addressed it with the ecospat framework [38,39]: a shared principal component analysis (PCA) was built from 10 continuous predictors (the 12-variable consensus set excluding the categorical land-cover layer) using both species’ occurrence points together with 10,000 random background locations, and each species’ occurrence density was projected onto a shared, gridded PC1-PC2 environmental space (100 × 100 grid, kernel density smoothed) following [38]. Niche overlap in this shared space was quantified with Schoener’s *D* and a modified Hellinger’s *I* statistic [40] and tested against two null models: a niche equivalency test (100 permutations of occurrence identity between species, asking whether the two niches are statistically indistinguishable) and a niche similarity test (100 randomizations of one species’ niche within the shared background, asking whether observed overlap exceeds that expected under random placement within the available environment) [41].

### 2.7. Joint Host Co-Occurrence Index

No georeferenced brucellosis case or livestock-outbreak data were available for this study region at a resolution suitable for disease mapping, so we constructed a joint host co-occurrence index (JCI) rather than a case-informed disease model; the index rests on the assumption, common in multi-host disease ecology, that opportunities for cross-species pathogen exchange are greater where candidate hosts overlap in space [42], an assumption that has not been tested for *Brucella* in this system. For each scenario, the JCI surface was calculated as the geometric mean of the two species’ continuous suitability rasters:

(1) $$joint\;risk=\sqrt{{suitability}_{squirrel}\times {suitability}_{pika}}$$

This requires both hosts to be at least moderately suitable to score highly (unlike an arithmetic mean, in which a high value for one host can compensate for a near-zero value in the other) while being less punitive toward moderate, non-zero co-suitability than a raw product. We adopt the geometric mean, rather than a density- or transmissibility-weighted combination, as the most conservative and transparent baseline available in the absence of species-specific data on relative population density, *Brucella* carriage rate, or contact frequency with livestock for either host; this choice is tested against stricter (minimum) and more lenient (arithmetic mean) alternatives in a sensitivity analysis below. A discrete host-overlap zone was additionally defined as the intersection of the two species’ binary suitable-habitat layers. The JCI is an ecological index of potential host overlap; it is not an epidemiological risk estimate, and a change in the JCI should not be read as a proportional change in brucellosis incidence or spillover.

### 2.8. Exploratory Transmission-Threshold Analysis (Next-Generation Matrix)

The JCI surface treats co-occurrence as a static snapshot; it does not ask whether a pathogen introduced where both hosts co-occur could actually sustain transmission. We therefore extended the analysis with an explicit epidemic-threshold model using the classic multi-host next-generation-matrix (NGM) framework [43,44,45,46,47]. No *Brucella* transmission-rate estimates exist for either host in this system, so absolute rates cannot be fitted; instead, each species’ MaxEnt suitability (already used as a relative-density proxy in the JCI analysis) stands in for local relative host density, and the within-species transmission coefficient is normalized to 1. The single remaining unknown, the ratio of cross-species to within-species transmission efficiency (*κ* = β_inter_/β_intra_), is not fixed at an assumed value but treated explicitly as a sensitivity axis (*κ* = 0.25, 0.5, 0.75, spanning weak to substantial cross-species transmission). For the symmetric 2 × 2 next-generation matrix with intraspecific entries equal to each species’ local suitability and cross terms equal to *κ* times the geometric mean of the two suitabilities, the dominant eigenvalue gives a spatially explicit basic reproduction number proxy:

(2) $$R_{0}\left(x\right)=\frac{N_{1}\left(x\right)+N_{2}\left(x\right)}{2}+\sqrt{{\left(\frac{N_{1}\left(x\right)-N_{2}\left(x\right)}{2}\right)}^{2}+\kappa^{2}\cdot N_{1}\left(x\right)\cdot N_{2}\left(x\right)}$$

computed for current climate and each future scenario at all three *κ* values, with *R*_0_ > 1 (the classic epidemic-threshold condition, above which an introduced infection is expected to spread rather than die out) mapped and its areal extent tracked across scenarios. This yields a mechanistically motivated, threshold-based complement to the purely statistical JCI, at the cost of remaining a relative proxy: it is silent on host population saturation, immunity, recovery, or the true absolute transmission rates, which are not measurable from habitat data alone. The value *R*_0_ = 1 is meaningful only relative to the scale of the within-species transmission coefficients, which were normalized to 1 rather than estimated; *R*_0_ is therefore treated as a dimensionless relative index, not as an estimate of epidemic potential or a validated epidemiological prediction. Suitability was used as a proxy for relative host density because it is the only spatially continuous quantity available for both species, on the assumption that habitat suitability is monotonically related to local abundance; this assumption can fail where competition, predation, grazing management or rodent control decouple density from habitat quality. To examine how much the results depend on these assumptions, we recomputed the area with *R*_0_ > 1 at *κ* = 0.5 after (i) raising suitability to the powers γ = 0.5 and 2 to represent saturating or accelerating suitability–density relationships, (ii) halving and doubling the pika’s within-species coefficient relative to the squirrel’s (ρ = 0.5 and 2), and (iii) halving and doubling both within-species coefficients (s = 0.5 and 2), with the generalized entries h1 = s·*N*_1_^γ^ and h2 = s·ρ·*N*_2_^γ^ replacing *N*_1_ and *N*_2_ in Equation (2).

### 2.9. Path Analysis of Climate, Suitability and JCI Change

To formally test how climate warming’s effect on joint host co-occurrence index is channeled through each species’ habitat suitability, we fitted a path model with lavaan [48] using grid cells pooled across the eight future scenarios as the sample unit (*n* = 3304 grid-cell × scenario observations, drawn from a target of 1500 regularly spaced candidate cells per scenario before masking to Inner Mongolia’s irregular boundary, which reduced the retained count to ~413 cells per scenario on average). For each cell and scenario, we computed the change (future–current) in: annual mean temperature (Δbio1, an exogenous general warming signal not itself used as a predictor in either species’ final MaxEnt model, and therefore independent of the suitability surfaces by construction), ground-squirrel suitability (ΔSuit_squirrel_), pika suitability (ΔSuit_pika_) and JCI (ΔJCI). The path model specified: ΔSuit_squirrel_ ~ Δbio1; ΔSuit_pika_ ~ Δbio1; ΔJCI ~ ΔSuit_squirrel_ + ΔSuit_pika_ + Δbio1, with standardized path coefficients (*a*_1_, *a*_2_ for the climate → suitability paths; *b*_1_, *b*_2_ for the suitability → JCI paths; *c* for the residual direct climate → JCI path) and indirect (mediated) effects (*a*_1_ × *b*_1_, *a*_2_ × *b*_2_) as the quantities of interest [27,28]. The model has one degree of freedom (the residual covariance between the two suitability changes was fixed at zero), and its fit was evaluated with χ^2^, CFI, TLI, RMSEA and SRMR; its standardized coefficients are comparable across the two host-specific pathways and describe whether climate-driven JCI change is associated more with the ground squirrel, the pika, or both.

Pooling grid cells across scenarios as independent observations risks anti-conservative standard errors if residuals are spatially autocorrelated, a common concern for raster-derived regressions [49,50]. We tested this directly with Moran’s *I* on the final-stage regression’s residuals, both for the full pooled sample and, to avoid the complication of the same locations recurring across scenarios, restricted to a single representative scenario. As a robustness check, we also refit the full path model on a far more sparsely and widely spaced subsample of cells per scenario (150 rather than 1500) to test whether the qualitative conclusions (coefficient signs and the relative magnitude of the two hosts’ suitability-to-JCI paths) survive greatly reduced spatial pseudoreplication. As the primary correction, we additionally refit both the first-stage (climate to suitability) and final-stage (suitability to JCI) equations as generalized least squares (GLS) models with an exponential spatial correlation structure on grid-cell coordinates, fitted separately by scenario with a nugget effect, giving standard errors and *p*-values that directly account for residual spatial dependence rather than assuming independence. Because the JCI is a deterministic function of the two suitability surfaces (Equation (1)), the suitability → JCI paths (b1, b2) are partly algebraic consequences of the index construction and are not treated as independent ecological mechanisms; the path model is used descriptively to show how climate-related change in each species’ suitability maps onto the JCI and not to infer causal mediation. Uncertainty was quantified as 95% confidence intervals for all standardized coefficients: for the pooled lavaan model by the delta method and for the spatial GLS models by refitting the three equations on z-scored variables and using the GLS standard errors, with the indirect effects (a1b1, a2b2) obtained by Monte Carlo propagation (20,000 draws) of the stage-wise sampling distributions of the coefficients.

### 2.10. Sensitivity to the JCI Combination Rule

Because the geometric-mean JCI formula is one of several defensible ways to combine two suitability surfaces, we tested whether the projected collapse in JCI is an artifact of that specific choice by recomputing landscape-mean JCI under two alternative rules for every scenario: the minimum of the two species’ suitabilities (a strict co-limitation rule, in which the JCI cannot exceed the less-suitable host) and the arithmetic mean (a lenient rule, in which high suitability in one host can partially compensate for low suitability in the other).

### 2.11. Background-Extent (M-Area) Sensitivity Check

To test directly whether restricting occurrence and background sampling to Inner Mongolia’s political boundary truncates the pika’s fitted climatic niche [51,52], we built an expanded environmental background covering Inner Mongolia plus a ~5-degree buffer into Mongolia and neighboring provinces (92.17–131.08 degrees E, 32.38–58.38 degrees N), using real WorldClim 2.1 bioclimatic and elevation data for the buffer zone and deriving slope and aspect from that elevation. No matching-provenance source was available for NDVI, human footprint, river distance or land cover in the buffer zone; these four layers were extended outward from the existing Inner-Mongolia-clipped rasters by nearest-value gap-filling, an explicit approximation rather than real external data, and a limitation of this check specifically. We refit the pika model with the same 12-variable consensus set using all 148 candidate occurrence points falling within this expanded extent (field survey plus GBIF, versus 17 within Inner Mongolia alone) and background sampled from the same extent, then projected the result back onto Inner Mongolia for direct comparison with the primary model. We subsequently extended this check to all eight future scenarios using real CMIP6 bioclimatic projections (MIROC6 general circulation model) for the same buffered extent, at 10 arc-minute resolution (~18.5 km, coarser than the ~4.6 km native resolution used elsewhere in this study, resampled to match the finer static-layer grid) to keep the download tractable; static (non-climate) layers were held at their current-day extended values for all future scenarios, consistent with the primary analysis’s own treatment of these layers. MIROC6 differs from the BCC-CSM2-MR model used for the primary analysis’s future scenarios; this cross-GCM difference is intentional rather than a confound to control for since this check is a directional robustness test of both the background extent and the choice of GCM together, not a like-for-like replacement of either alone.

### 2.12. Robustness Analyses of the Pika Projections and Climate-Model Uncertainty

Because the projected loss of pika habitat is a central finding that rests on only 17 occurrence records, we tested its robustness in five ways (analysis code: 26_pika_robustness.R). First, we quantified spatial clustering and environmental representation of the records: nearest-neighbor distances, the Clark–Evans ratio (R < 1 indicates clustering), the number of clusters at a 100 km cut-off, and the share of Inner Mongolia grid cells lying within the range of each climate predictor sampled by the records. Second, we refitted the pika model with alternative specifications: linear + quadratic (LQ) features with regularization multipliers (RM) of 1, 2 and 4, linear (L) features only, a model excluding bio8, and a model using only the five climate predictors (bio2, bio3, bio8, bio14, bio15), which removes dependence on the non-climatic layers held constant in the future. Third, we changed the occurrence data by spatial thinning to minimum separations of 10, 20 and 40 km and by 50 random subsamples of 12 of the 17 records; refitted models otherwise used the primary settings (10,000 background points, 10th-percentile threshold). Fourth, we recalculated the habitat loss of the primary model with alternative binary thresholds (minimum training presence, the 10th and 20th percentiles of training presences, and the threshold maximizing the sum of sensitivity and specificity, MaxSSS) with a threshold-free metric (landscape-mean suitability) and with all non-climatic layers held at current-day values in every future scenario. Fifth, to examine climate-model uncertainty, we applied the primary pika and squirrel models to MIROC6 climate projections for the same eight scenarios (10 arc-minute data resampled to the 4.6 km grid; climate predictors replaced, all other layers unchanged), and we calculated the share of future grid cells in which bio8 and the five climate predictors jointly lay outside the range sampled by the pika records, as an indicator of extrapolation.

### 2.13. Statistical Software

All spatial data processing, statistical modeling and visualization were performed in R (version 4.6.0; R Foundation for Statistical Computing, Vienna, Austria) using the following packages, obtained from the Comprehensive R Archive Network (CRAN) unless noted: terra (v1.9-46) and tidyterra (v1.3.0) for raster and vector geoprocessing; maxnet (v0.1.4) for MaxEnt model fitting; ENMeval (v2.0.5.2) [24] for hyperparameter tuning; usdm (v2.1.7) [35] for variance-inflation-factor variable screening; sf (v1.1.2) and ggspatial (v1.1.10) for spatial data handling and map annotation; ecospat (v4.1.4) [39] for niche-overlap analysis; lavaan (v0.7.2) [48] for path/mediation modeling; nlme (v3.1-169) for spatial generalized least squares regression; ape (v5.8.1) for Moran’s *I* spatial-autocorrelation testing; and ggplot2 (v4.0.3), ggalluvial (v0.12.6), patchwork (v1.3.2), scales (v1.4.0), viridis (v0.6.5) and dplyr (v1.2.1) for data manipulation and figure production. Ground squirrel field-survey coordinates were recorded with handheld GPS receivers (GPSMAP H1; Garmin Ltd., Olathe, KS, USA; WGS84 datum) as part of the source rodent-survey dataset; pika occurrence records were compiled from existing field-survey and GBIF data [30] rather than newly collected for this study. No laboratory assays, reagent kits, or biological sampling of any kind were performed for this work: all analyses are computational, built entirely from pre-existing occurrence records and publicly available environmental raster datasets, consistent with the non-invasive, observational study design (see Institutional Review Board Statement). Analysis code is available on request (Data Availability Statement).

## 3. Results

### 3.1. Variable Selection and Model Performance

The three-method screening procedure reduced the 26-variable candidate pool to an identical 12-variable consensus set for both species (Figure 2). The ENMeval-tuned model for *Spermophilus dauricus* selected a hinge-only feature class with regularization multiplier = 0.5 (training AUC = 0.896; validation AUC = 0.808 ± 0.080; continuous Boyce index = 0.565), with NDVI (29.0%), isothermality (bio3, 16.6%), elevation (14.3%), precipitation seasonality (bio15, 11.6%) and human footprint (9.7%) as the leading predictors (Figure 3a and Figure 4a). The model for *Ochotona dauurica* selected a linear + quadratic feature class with regularization multiplier = 4 (training AUC = 0.957; leave-one-out validation AUC = 0.940 ± 0.251), dominated by mean temperature of the wettest quarter (bio8, 57.9%) and NDVI (30.9%), with isothermality (bio3, 6.6%) and precipitation of the driest month (bio14, 2.6%) contributing modestly (Figure 3b and Figure 4b). The response curve for bio8 showed a steep sigmoidal decline in predicted pika suitability once mean wettest-quarter temperature exceeded ~18–19 °C (Figure 3b). The continuous Boyce index could not be computed reliably for the pika model given the very small leave-one-out sample; this, together with the wide standard deviation on validation AUC, reflects the reduced statistical power of the *n* = 17 pika dataset.

The leave-one-out refitting check re-estimated the pika’s bio8 threshold 17 times, once per left-out occurrence point: the suitability = 0.5 crossing fell within a narrow 19.16–19.55 °C band (mean 19.4 °C, SD = 0.12 °C) across all 17 refits, and the full-sample estimate (19.27 °C) sits inside that range.

### 3.2. Current Habitat Suitability

Under current climate, predicted suitable habitat for *S. dauricus* totaled 301,371 km^2^, broadly distributed across the central and eastern grassland belt with the highest suitability in a corridor spanning roughly 110–118° E (Figure 5a). Predicted suitable habitat for *O. dauurica* was smaller (136,458 km^2^) and more spatially concentrated, with the single highest-suitability patch located in northwestern Hulunbuir near 116–118° E, 49–50° N, and a secondary high-suitability zone in the north-central grassland near 112–116° E, 44–45° N (Figure 5b). The two species’ current high-suitability zones overlap substantially in the central-eastern grassland corridor, yielding a current joint host co-occurrence zone of 66,737 km^2^.

### 3.3. Interspecific Environmental Niche Overlap

In the shared PCA-environment space (PC1-PC2 explaining 48.7% of variance across the 10 continuous predictors), the two species showed moderate niche overlap: Schoener’s *D* = 0.462 and Hellinger’s *I* = 0.615 (Figure 6). The niche equivalency test did not reject the null hypothesis that the two niches are equivalent (*D* = 0.462, *p* = 0.772), so there is no statistical evidence that the two niches differ, although the small pika sample (*n* = 17) limits the power of this test. The niche similarity test, in contrast, rejected the null hypothesis that this level of overlap could arise from either species occupying a random position within the shared regional environmental background (*D* = 0.462, *p* = 0.010): the two species’ niches are significantly more similar to each other than chance placement within the available environment would predict. Read together, the two tests indicate that *S. dauricus* and *O. dauurica* are drawn toward a shared region of environmental space (consistent with both favoring open grassland conditions over the full range of habitat available in Inner Mongolia). The equivalency test did not separate them, although *O. dauurica*’s occupied niche (Figure 6, right) appears narrower and more concentrated than *S. dauricus*’s (Figure 6, left). This independent, environment-space measure of niche relationship agrees with the geographic co-occurrence pattern: the two species are ecologically similar enough to plausibly share habitat.

### 3.4. Projected Range Change Under Future Climate

Projected suitable area declined for both species under every future scenario, but with markedly different magnitudes (Figure 7; Table 1). For *S. dauricus*, suitable area declined by 33.1–48.0% by the 2050s and by 9.6–84.3% by the 2070s, with the smallest loss under SSP1-2.6 (2070s) and the largest under SSP5-8.5 (2070s), and the per-cell suitability-change distributions in Figure 7b show this loss concentrated in the lower tail rather than a uniform shift. For *O. dauurica*, losses were far more severe and essentially saturated regardless of emissions pathway: suitable area collapsed by ≥99.9% under every scenario, falling to exactly zero mapped suitable cells under 2070s SSP5-8.5; the least extreme of the eight declines were 2050s SSP2-4.5 (−99.96%) and 2070s SSP1-2.6 (−99.92%). This pattern is consistent with the pika model’s strong dependence on mean temperature of the wettest quarter (bio8): the threshold in the response curve (~18–19 °C) is exceeded across nearly the entire study area under every future scenario. Suitability-weighted centroid shifts followed the same pattern, though a meaningful bootstrap estimate (Figure 7c) could only be computed for the three pika scenarios retaining more than one suitable grid cell, where the pika centroid shifted by up to 760 km on average (bootstrap mean ± SD, 2050s SSP1-2.6), several times farther than the ground squirrel’s largest bootstrap-mean shift (163 km, 2050s SSP2-4.5). In the remaining five scenarios, pika suitable habitat collapsed to a single residual grid cell (Table 1), for which bootstrap resampling is degenerate (zero variance); the point centroid shift for these scenarios was a consistent 1031 km, reflecting collapse to the same peripheral cell each time, and these scenarios are accordingly omitted from Figure 7c.

### 3.5. Joint Host Co-Occurrence Index

The JCI surface contracted even more sharply than either species’ individual range since it requires both hosts to be simultaneously suitable (Figure 8; Table 1). The discrete host-overlap zone declined from 66,737 km^2^ under current climate to at most 32 km^2^ under any future scenario (an area change of −99.95% to −100%), with six of the eight scenarios (2050s SSP3-7.0/SSP5-8.5 and all four 2070s scenarios) leaving no discrete overlap cells at all.

### 3.6. Exploratory Transmission-Threshold Analysis

The exploratory NGM *R*_0_ index showed the same direction of change as the JCI under an epidemic-threshold framing (Figure 9). Under current climate, the area exceeding the *R*_0_ = 1 threshold ranged from 29,379 km^2^ at the most conservative cross-species transmission assumption (*κ* = 0.25) to 94,803 km^2^ at the most permissive (*κ* = 0.75), concentrated in the same central-eastern grassland corridor identified by the JCI surface (Figure 9a). This area contracted under nearly every future scenario and *κ* combination, by 19–97% depending on scenario and *κ*, mirroring the JCI pattern. The one exception was the mildest scenario (2070s SSP1-2.6) at the most conservative transmission assumption (*κ* = 0.25), where the *R*_0_ > 1 area increased by 20.9% relative to current, a divergence from the otherwise uniform decline that arises because at low *κ*, the NGM eigenvalue is driven predominantly by whichever host has the higher local suitability rather than requiring both hosts jointly, and the ground squirrel’s suitability itself increases under this specific mild-warming, long-horizon combination.

To test how much these results depend on the parameterization, we recomputed the area with *R*_0_ > 1 at *κ* = 0.5 under alternative assumptions (Table 2). Changing the suitability–density relationship (γ = 0.5 or 2) or doubling either within-species coefficient left the direction of change unchanged, with declines of 11–99.5% in every scenario. Halving the pika’s within-species coefficient (ρ = 0.5), however, reduced the current area from 64,033 to 17,637 km^2^ and produced increases of 3.9–138.6% in four of the eight scenarios, and halving both within-species coefficients (s = 0.5) eliminated the area with *R*_0_ > 1 altogether, because *R*_0_ never exceeded 1. The absolute extent of the *R*_0_ > 1 area, and whether it increases or decreases in some scenarios, therefore depends on assumed coefficients that cannot be estimated from these data, and the *R*_0_ results should be read as a sensitivity exploration of a relative index, not as a validated epidemiological prediction.

### 3.7. Path Analysis of Climate, Suitability and JCI Change

The path model (*n* = 3304 pooled grid-cell × scenario observations; Figure 10) showed that climate anomaly (Δbio1) was a significant predictor of suitability change for both species, but nearly three times more strongly for *S. dauricus* (standardized β = −0.195, *p* < 0.001) than for *O. dauurica* (β = −0.059, *p* = 0.001). This asymmetry is consistent with the two species’ response-curve shapes (Figure 3): the ground squirrel’s suitability responds relatively smoothly to a general warming signal, whereas the pika’s suitability is governed by a sharp threshold in a specific seasonal variable (bio8) that a generic annual-mean-temperature anomaly captures only partially. In the reverse direction, however, suitability change was a substantially stronger predictor of JCI change for the pika (β = 0.635, *p* < 0.001) than for the ground squirrel (β = 0.324, *p* < 0.001), nearly twice the standardized effect (although these suitability → JCI paths follow in part from how the JCI is calculated). The standardized indirect (mediated) effect of climate anomaly on JCI was slightly larger through the ground-squirrel pathway (a1 × b1 = −0.063) than through the pika pathway (a2 × b2 = −0.038), while the residual direct climate → JCI path was small but significant (*c* = 0.117, *p* < 0.001) and the total effect was not significantly different from zero (β = 0.016, *p* = 0.357), reflecting the net cancellation of a general linear warming signal against the highly non-linear, threshold-driven collapse in pika suitability that a linear path model can only partially capture.

Because grid cells pooled across scenarios are spatially structured, we tested the final-stage regression’s residuals for spatial autocorrelation with Moran’s *I*: both the full pooled model (*I* = 0.230, *p* < 0.001, one representative scenario’s locations) and a single-scenario-only fit (*I* = 0.142, *p* < 0.001) showed significant positive spatial autocorrelation. The *p*-values reported above should therefore be read as descriptive rather than strictly confirmatory; point estimates remain informative, but their nominal significance is likely overstated. As a robustness check, we refit the full path model on a far more sparsely spaced subsample (a target of 150 regularly spaced cells per scenario before masking, versus 1500 in the primary analysis; the same irregular-boundary masking that reduced the primary sample also applied here, yielding 49 retained cells per scenario, *n* = 392 total), which reduced residual spatial autocorrelation substantially (Moran’s *I* = 0.092, still significant but roughly 35–60% smaller than in the denser samples). Under this much more spatially conservative subsample, the central qualitative finding was preserved and, if anything, strengthened: the pika’s suitability-to-JCI path (β = 0.680) remained substantially larger than the ground squirrel’s (β = 0.268), a wider gap than in the primary analysis. The climate-to-pika-suitability path lost formal significance at this reduced sample size (β = −0.069, *p* = 0.169), consistent with reduced statistical power rather than a qualitatively different effect since its point estimate and sign were essentially unchanged from the primary analysis.

A spatial generalized least squares (GLS) model with an exponential spatial correlation structure (fitted range ≈ 5.5 grid cells, nugget = 0.37) gave the same qualitative picture as the thinned-sample check on a third, independently sampled subset (target of 200 regularly spaced cells per scenario before masking, retaining *n* = 536 cells total, larger than the thinned check but still far short of the primary analysis’s *n* = 3304 since a dense spatial correlation matrix is computationally intractable at the full sample size). The two suitability-to-JCI paths remained highly significant and preserved their relative ordering (unstandardized coefficients; pika: 0.455, *p* < 0.001; ground squirrel: 0.227, *p* < 0.001; standardized values with confidence intervals are given in Table 3), indicating that the pika’s roughly two-fold larger coefficient for JCI change is not an artifact of spatial pseudoreplication. The residual direct climate → JCI path, significant under naive pooled OLS (*p* < 0.001), was no longer significant once spatial dependence was properly modeled (*p* = 0.134), indicating that association was partly an artifact of spatial autocorrelation rather than a robust direct effect. In the first-stage equations, the climate → squirrel-suitability path remained significant under GLS (*p* = 0.004), but the climate → pika-suitability path did not (*p* = 0.626), even though its point estimate retained the same sign as in the naive analysis.

Table 3 gives 95% confidence intervals for the standardized coefficients and indirect effects. In the pooled model, all paths other than the total effect were significant, but under the spatial GLS, the climate → pika-suitability path (β = −0.061; 95% CI −0.305 to 0.184) and the direct climate → JCI path (β = 0.132; −0.040 to 0.305) were statistically indistinguishable from zero. The indirect effect through the squirrel remained negative with an interval excluding zero (−0.046; −0.081 to −0.014), whereas the indirect effect through the pika (−0.037; −0.185 to 0.109) and the total indirect effect (−0.083; −0.235 to 0.066) did not. The suitability → JCI paths were large under both estimators (pika 0.601 [0.545, 0.657] and squirrel 0.256 [0.206, 0.307] under the GLS) but, because the JCI is calculated from the two suitability surfaces, they mainly reflect the index construction. The pooled model had one degree of freedom and an imperfect fit (χ^2^(1) = 31.9, CFI = 0.987, TLI = 0.921, RMSEA = 0.097, SRMR = 0.040).

### 3.8. Sensitivity to the JCI Combination Rule

The projected collapse in the joint host co-occurrence index was not an artifact of the geometric-mean formula: landscape-mean JCI declined under every future scenario regardless of combination rule, by 69.2–97.9% under the geometric mean, 85.9–99.5% under the stricter minimum rule, and 52.7–92.0% under the more lenient arithmetic mean. The minimum rule produced the steepest declines, as expected since it admits no compensation between hosts, while the arithmetic mean produced the shallowest. The three rules agreed on both the qualitative pattern (severe decline under every scenario, including the mildest, SSP1-2.6) and the ordering of scenarios by severity. The direction and near-universality of the JCI collapse is therefore robust to how the two species’ suitabilities are combined, even though its exact magnitude is not.

### 3.9. Background-Extent (M-Area) Sensitivity Check

The expanded-background refit showed that restricting training to Inner Mongolia underestimated the pika’s current suitable area; when extended to all eight future scenarios with real CMIP6 data, however, the central conclusion was robust to this restriction. Current suitable area within Inner Mongolia increased from 136,458 km^2^ under the primary (Inner-Mongolia-only) model to 213,485 km^2^ under the expanded-background model, a 56% increase, confirming that restricting training to Inner Mongolia’s political boundary meaningfully underestimated the pika’s absolute climatic tolerance, and the sharp bio8 suitability threshold that anchored the primary model’s response curve did not reproduce as a single clean 0.5-crossing once the broader climatic range accessible to the species was included in training. However, projecting this expanded-background model onto all eight future scenarios showed that the severity of future decline itself is not an artifact of this truncation: suitable area within Inner Mongolia collapsed by 98.7–100% under the expanded-background model (falling to zero under both 2070s SSP3-7.0 and SSP5-8.5), closely tracking the 99.9–100% collapse projected by the primary model across the same eight scenarios (Figure 11e). In absolute terms, the expanded model consistently projected more residual suitable area than the primary model in every scenario where any remained (e.g., 2711 km^2^ versus 17 km^2^ under 2050s SSP3-7.0), consistent with its larger current-climate baseline, but the proportional severity of the projected decline was essentially unchanged.

### 3.10. Robustness of the Pika Projections and Climate-Model Uncertainty

The 17 pika records were strongly clustered (nearest-neighbor distance median 12.5 km and mean 49.1 km; Clark–Evans *R* = 0.378; five clusters at a 100 km cut-off) and were located in only four of the 12 prefecture-level units. They also sampled a narrow part of the regional climate: the records spanned 16.40–19.12 °C in bio8, a range that includes only 32.0% of present-day Inner Mongolia grid cells, and only 12.0% of cells fall within the sampled range of all five climate predictors simultaneously. For bio8, 53.5% of present-day cells already exceed the highest value recorded at a pika record (19.12 °C), and this share rises to 91.5–100% of cells in the eight future scenarios (Figure 12).

The near-total loss of pika habitat was robust to most changes in model specification and occurrence data (Table 4). With LQ features and regularization multipliers of 1, 2 and 4, suitable area declined by 99.3–100% in all eight scenarios; spatial thinning to 10, 20 and 40 km (14, 11 and eight records) gave declines of 99.4–100%; and in 48 of 50 random subsamples of 12 records, all eight scenarios declined by at least 90% (40 of 50 by at least 99%). The result was not independent of model structure, however. A linear-only model produced smaller declines (72.9–99.5%, with only one scenario at or above 99%), and a model excluding bio8 projected an increase in suitable area in every scenario (+54.9% to +151.7%), so the projected collapse is attributable to the response to bio8. A model using only the five climate predictors gave declines of 98.9–100%, and holding all non-climatic layers at current-day values left the declines of the primary model unchanged (99.9–100%), so the result does not depend on the non-climatic layers held constant in the future.

The magnitude of the decline was insensitive to the binary threshold: with the minimum training presence (identical to the MaxSSS threshold), the 10th-percentile and the 20th-percentile thresholds, current suitable area was 196,597, 136,458 and 71,993 km^2^, respectively, but the projected declines were 99.9–100% in every case, and landscape-mean suitability, a threshold-free metric, declined by 91.8–99.6%. Under MIROC6 climate projections, the primary pika model again projected a 100% loss of suitable area in all eight scenarios, as did the overlap of the two species (99.95–100% under BCC-CSM2-MR), whereas the ground squirrel model projected declines of 35.7–79.1% (compared with 9.6–84.3% under BCC-CSM2-MR); the scenario with the smallest squirrel loss differed between the two climate models (2070s SSP1-2.6 under BCC-CSM2-MR and 2050s SSP1-2.6 under MIROC6).

## 4. Discussion

A joint host co-occurrence surface is only as informative as the two underlying niche models are reliable, and it is only useful for surveillance planning if it says something that a single-species model cannot. Both conditions are met only in part: both models achieved acceptable-to-good discrimination on held-out data (validation AUC > 0.8), although the pika estimate is highly uncertain (±0.251); the variables selected are ecologically consistent with each species’ known habitat associations [9], and the resulting joint surface, together with the path model built on top of it, yields a conclusion that neither species’ map alone would reveal, namely that the two hosts are not equally associated with changes in where their suitable habitats overlap under warming.

### 4.1. Variable Selection and Model Performance

The predictors selected for each species (vegetation productivity (NDVI) and thermal seasonality (isothermality, bio3) for *Spermophilus dauricus*; summer temperature (bio8) and NDVI for *Ochotona dauurica*) are ecologically consistent with each species’ known habitat associations [9]: ground squirrels forage on green vegetation across the open steppe and tolerate a wide range of seasonal temperature swings, whereas pikas rely on cooler, moister microhabitats and are physiologically constrained by summer heat, which is exactly the seasonal window bio8 captures. This plausibility holds even though the two models had to be built differently: the squirrel model, with *n* = 620 occurrences, could support a flexible hinge-only feature class, whereas the pika model, with only *n* = 17, was deliberately restricted to linear and quadratic terms and evaluated by leave-one-out rather than k-fold cross-validation. That the pika’s dominant response curve nonetheless converges on a narrow, repeatable threshold (the suitability = 0.5 crossing varied by only 0.4 °C across all 17 leave-one-out refits) argues that this threshold is a genuine feature of the occurrence data rather than an artifact of any single point, even though it does not by itself resolve the broader statistical-power problem that a sample of 17 presences (below general recommended minima for reliable MaxEnt fitting [36]) necessarily carries; the wide standard deviation on validation AUC and the non-computable Boyce index for this species are symptoms of the same underlying limitation.

### 4.2. Current Habitat Suitability

The current-climate suitability surfaces place both species’ highest-suitability areas in the same central-eastern grassland corridor, which is also where Inner Mongolia’s grazing economy is most concentrated; this spatial coincidence is consistent with why the region has historically reported some of the country’s highest human brucellosis incidence [1], with incidence itself showing fine-scale spatial clustering tied to the pastoral landscape [53,54] and with a sylvatic-reservoir hypothesis (untested, because infection data are lacking) in which both candidate hosts are most available to livestock and herders in exactly the area where contact opportunities are already highest. The pika’s current distribution is more spatially concentrated than the squirrel’s, with its single highest-suitability patch in northwestern Hulunbuir, a pattern that, combined with its narrower environmental niche, foreshadows its disproportionate future vulnerability, consistent with field evidence that Daurian pika abundance is itself highly sensitive to local habitat and forage conditions [55,56,57].

### 4.3. Interspecific Environmental Niche Overlap

The two niche-overlap tests describe a coherent ecological relationship: *S. dauricus* and *O. dauurica* are drawn toward a shared region of environmental space (consistent with both favoring open grassland conditions over the full range of habitat available in Inner Mongolia), while the equivalency test could not separate them statistically, possibly because of the small pika sample. Such overlap is compatible with a joint range that is highly sensitive to whichever species’ narrower tolerance is exceeded first under warming. The pika’s occupied niche appears narrower and more concentrated than the squirrel’s (Figure 6), foreshadowing the asymmetric range contraction projected for the two species and independently corroborating, in the environment space, the geographic collapse of the JCI.

### 4.4. Projected Range Change Under Future Climate

The central finding of this study is an asymmetric change, not a simple parallel decline. The ground squirrel’s suitable habitat declined moderately and comparatively linearly with warming, consistent with its status as a habitat generalist tolerant of a broad climatic envelope. The pika, in contrast, showed near-total habitat collapse under almost every future scenario, a pattern that, in the fitted models, follows from the steep bio8 suitability threshold, which a linear warming proxy captures only weakly (Section 4.10 shows how strongly the result depends on this variable). This mirrors well-documented climate vulnerability in other *Ochotona* species, most notably range contraction and local extirpation in the American pika (*O. princeps*) attributed to thermal intolerance [15,58,59], consistent with the low upper thermal tolerance and narrow behavioral thermoregulation documented physiologically in the genus [60], and suggests that Daurian pika populations in Inner Mongolia may be similarly exposed. It is also consistent with a broader pattern in which climate warming is reshaping small-mammal community composition on the Mongolian Plateau [61,62], as well as species ranges more generally [63,64]. The centroid-shift asymmetry reinforces this picture: where a bootstrap estimate could be computed at all, pika range centroids moved several times farther than the squirrel’s, and in the majority of future scenarios, pika suitable habitat contracted to so few residual cells that a bootstrap distribution could not be meaningfully computed in the first place, itself a signal of how close to total collapse the projections are.

### 4.5. Joint Host Co-Occurrence Index

The joint co-occurrence surface contracted more sharply than either species’ individual range on its own because it is bounded by whichever host is locally less suitable at a given point: a cell only registers a high JCI if both hosts are simultaneously present, so a decline in either host alone is sufficient to erode it. Given this asymmetry, this near-total collapse is therefore, in the fitted models, driven predominantly by the pika’s steep climate sensitivity rather than by the comparatively moderate decline projected for the ground squirrel; the joint surface effectively inherits the fragility of its more vulnerable member. This is the geographic expression of the same asymmetry that the path model quantifies statistically.

### 4.6. Exploratory Transmission-Threshold Analysis

The transmission-threshold extension adds a threshold framing to the JCI rather than a second, independent line of evidence: because the NGM formulation is built directly from the same two suitability surfaces, its *R*_0_ > 1 contraction necessarily tracks the JCI contraction. Its value lies in reframing the same spatial pattern in terms of a persistence threshold rather than merely a co-occurrence score, while acknowledging that the threshold is defined relative to assumed, uncalibrated coefficients and in exposing how sensitive that reframing is to cross-species transmission efficiency, a parameter that this system’s data cannot currently constrain and that determines, more broadly, whether a system behaves as a set of coupled single-host epidemics or a genuinely joint one [65,66,67,68,69]. The single scenario in which the *R*_0_ > 1 area increased (2070s SSP1-2.6 at the lowest transmission-efficiency setting, *κ* = 0.25) is a negative case rather than a modeling artifact: at low *κ*, the NGM eigenvalue is driven predominantly by whichever host has the higher local suitability rather than requiring both hosts jointly, and the ground squirrel’s own suitability happens to increase under this specific mild-warming, long-horizon combination. It shows that the pika-driven collapse conclusion holds across most, but not all, of the explored parameter space, and that resolving whether cross-species transmission is closer to weak or substantial is an important priority for future serological or contact-rate studies. The sensitivity analysis (Table 2) shows that the absolute extent of the area with *R*_0_ > 1, and even the sign of its change in some scenarios, depends on the assumed pika coefficient and on the scale of the within-species coefficients; *R*_0_ values and threshold areas should therefore not be interpreted as epidemiologically estimated quantities, and the main use of this analysis is to identify which unmeasured parameters (cross-species transmission efficiency, relative host competence and the density–suitability relationship) most need empirical estimates.

### 4.7. Path Analysis of Climate, Suitability and JCI Change

Taken together, the path coefficients describe an asymmetric pattern: mean warming is a better linear predictor of the squirrel’s habitat trajectory, but once habitat change occurs, it is the pika’s suitability that exerts substantially greater leverage over the JCI surface, nearly double the standardized weight of the squirrel’s pathway. Because the JCI is a deterministic function of the two suitability surfaces, the b-paths follow in part from how the index is constructed; they should not be read as independent ecological mechanisms or as causal mediation. For ecological monitoring, this asymmetry suggests that pika distribution shifts are the more informative indicator of change in the area where the two candidate hosts overlap, whereas the ground squirrel’s decline is more readily tracked with coarse climate indicators. For epidemiological surveillance, however, the path model offers no guidance on which species contributes more to *Brucella* maintenance or livestock spillover because infection status, population density and contact rates with livestock are unknown for both species; surveillance priorities should therefore be set with serological and contact-rate data, not by the relative size of the path coefficients. The spatial diagnostics qualify this picture [50,70,71]: under the spatially corrected GLS model, the climate → pika-suitability path (standardized β = −0.061, 95% CI −0.305 to 0.184) and the indirect effect through the pika (−0.037, −0.185 to 0.109) are statistically indistinguishable from zero, whereas the corresponding squirrel paths are not (a1 = −0.181, −0.304 to −0.058; indirect effect −0.046, −0.081 to −0.014). The climate signal is therefore transmitted to the JCI more reliably through the squirrel than through the pika, even though the pika’s suitability → JCI coefficient is larger. This is consistent with the pika’s response operating through a sharp threshold in a specific seasonal variable rather than through general warming, but it also shows that the pika’s role as the main correlate of JCI change is a statement about the index construction and the size of the projected suitability change, not about an independently established mechanism; path/SEM coefficients are best read as descriptions of relative association rather than causal effects [72].

### 4.8. Sensitivity to the JCI Combination Rule

That the collapse in JCI holds regardless of whether hosts are combined by geometric mean, strict minimum, or lenient arithmetic mean is an important robustness result: these three rules encode genuinely different assumptions about how the two hosts’ suitabilities jointly determine the index (full co-limitation, partial compensation, and complete compensation, respectively), and a conclusion that depended on which of these biologically distinct assumptions was chosen would be far more fragile than one that does not. Because the direction and near-universality of the collapse is insensitive to this choice, the uncertainty that remains genuinely open is the exact magnitude of the JCI decline, not whether a decline occurs at all.

### 4.9. Background-Extent (M-Area) Sensitivity Check

The expanded-background refit shows that restricting training to Inner Mongolia’s political boundary underestimated the pika’s absolute climatic tolerance, consistent with the general finding that background/accessible-area choice measurably biases estimated niche breadth [51,52]: current suitable area increased by 56% once the background extended into Mongolia and neighboring provinces, and the sharp bio8 threshold that anchored the primary model’s response curve no longer reproduced as a single clean 0.5-crossing once this broader climatic range was included in training. However, projecting the expanded-background model onto all eight future scenarios shows that the severity of the projected future decline is not an artifact of this truncation: suitable area collapsed by 98.7–100% under the expanded-background model, closely tracking the 99.9–100% collapse projected by the primary model across the same eight scenarios. The near-total collapse projected for the pika is therefore not an artifact of restricting the background to Inner Mongolia, although the absolute current-climate area estimates are. The boundary-truncation concern is not equally acute for both species: ground squirrel occurrences sit well within the interior (median 95.7 km from the border), while pika occurrences sit considerably closer to it (median 42.7 km), so this check specifically targets the species for which it matters most.

### 4.10. Robustness of the Pika Projections and Climate-Model Uncertainty

The 17 pika records are a small, spatially clustered and climatically restricted sample, and each of these properties can bias projections. Clustering (Clark–Evans *R* = 0.378; five clusters) means that the records represent fewer independent localities than their number suggests, so the fitted bio8 response is effectively constrained by a handful of sites. The records were not collected by a design covering the species’ range, and no target-group background or bias layer was available, so sampling bias toward surveyed areas cannot be excluded. The records cover 16.4–19.1 °C in bio8, and only 12% of present-day grid cells fall within the sampled range of all five climate predictors; the model therefore has to infer the species’ response outside the sampled climate from the contrast with background points, and the future climates of Inner Mongolia (bio8 above the highest recorded value in 91.5–100% of cells; Figure 12) lie almost entirely beyond the sampled range. Predictions in that region depend on the shape of the quadratic term and are extrapolations.

Within these limits, the projected loss is robust in some respects and not in others (Table 4). It was insensitive to regularization, spatial thinning, random removal of records, the binary threshold, the choice of non-climatic layers and the climate model, which suggests that it is not an artifact of any single record, of clustering at the scales tested, or of the 10th-percentile rule. It was, however, entirely dependent on the temperature response: a model without bio8 projected an increase in suitable area, and a linear-only model projected a markedly smaller loss (72.9–99.5%), so the quadratic decline in suitability above about 19 °C is the sole source of the near-total collapse. Because the sampled range ends at 19.1 °C, while Inner Mongolia’s present-day bio8 distribution already extends well above it, the model treats warmer cells as unsuitable because no records occur there, which is a plausible but untested inference about the species’ thermal limit. The projected collapse should therefore be read as the outcome of one statistically stable but extrapolated response, not as a measured result; we regard its direction as more reliable than its magnitude, and we recommend testing it with independent occurrence data from warmer parts of the species’ range, particularly from Mongolia, where the expanded-background analysis (Section 3.9) found a 56% larger current suitable area.

The use of a single climate model was addressed by repeating the projections with MIROC6, which gave the same qualitative result for the pika and for the overlap (100% loss) and a similar range of squirrel declines (35.7–79.1%), although the scenario ranking of squirrel loss differed. This comparison is limited to two models, one of them at coarse resolution, and does not span the full uncertainty in precipitation projections for semi-arid Inner Mongolia. Likewise, the choice of binary threshold changed the current suitable area by a factor of 2.7 (71,993–196,597 km^2^) but not the relative loss, and holding non-climatic layers at current values did not change the results, so the uncertainty about the magnitude of loss lies in the temperature response and in extrapolation rather than in thresholding or static layers.

### 4.11. Implications for Livestock and Human Brucellosis and a One Health Perspective

Human brucellosis in China is acquired mainly from infected livestock, chiefly sheep, goats and cattle [1,3], and the spatial pattern of human cases in Inner Mongolia has been linked to environmental and livestock-related factors [53,54]. Any consequence of the projected host shifts for livestock or human infection would therefore have to run through contact between wildlife and grazing livestock, which this study did not measure: the JCI shows where the two candidate hosts are expected to overlap, and we did not overlay it on livestock density, herd serology or human cases. Plausible but untested pathways include livestock contact with pasture, water or burrow areas contaminated by infected wildlife, and direct contact of herders with animals or carcasses; we have no evidence that either species is infected, so these remain hypotheses.

Which of the two species matters more for transmission to cattle and humans cannot be answered from the available evidence. For *S. dauricus*, a plague-surveillance paper mentions brucellosis among the diseases the species may transmit but gives no data [73]; for pikas, the only figure is a review’s 0.78% seropositivity for unnamed “pikas” [74]; and related taxa show that ground squirrels can retain *Brucella* experimentally [75] and that marmots can be seropositive and yield isolates in the field [76]. On ecological grounds alone, the ground squirrel is the more likely interface with livestock because it is more widespread (301,371 km^2^ versus 136,458 km^2^ of currently suitable habitat) and much less climate-sensitive, but this is an inference about opportunity for contact, not about infection or competence. Resolving the question requires serological and molecular testing of both species sampled together with livestock.

The question of what follows if the overlap zone shrinks substantially cannot be answered by habitat models alone, and the direction is not necessarily protective. If wildlife contribute little to livestock infection, the loss of overlap changes little, because transmission is then maintained within and between livestock and by human practices. If one or both hosts do contribute, the effect of losing a host depends on its relative contribution: removing a highly competent host can reduce transmission, whereas removing a less competent one can leave it unchanged or increase it, as general multi-host theory shows [65,66,67]. Habitat loss also does not remove animals at once; remnant populations may concentrate in refugia, where higher local density could increase contact among hosts and with livestock, which we did not test. The projected change is also asymmetric: the squirrel retains 15.7–90.4% of its current suitable area, whereas the pika loses almost all of it, so a squirrel–livestock interface would persist across much of the squirrel’s range under most scenarios while any pika–livestock interface would largely disappear. The NGM exploration points the same way but is assumption-dependent (Table 2). These results describe how the geography of potential hosts may shift, not whether the number of human or livestock cases would rise or fall.

Brucellosis is a One Health problem in the usual sense: human cases arise from animal reservoirs, control in livestock (vaccination, testing and culling, movement control) is the main lever for human disease [3], and wildlife and environmental factors, here climate and grazing, may modify the system at its edges. Within that framing, the present study supplies a single layer, the projected geography of two candidate wildlife hosts, and shows that this layer is itself uncertain (Section 4.10). Linking it to animal and human health needs data from the other sectors: (i) serological and molecular testing of both species at sites chosen along the JCI gradient, sampled together with livestock herds and, where possible, human cases from the same counties; (ii) livestock-density and grazing-intensity layers [77,78] to weight host co-occurrence by exposure; and (iii) shared reporting among veterinary, public-health and wildlife or forestry agencies. Because *S. dauricus* is already trapped in plague surveillance in northern China [73], adding *Brucella* testing to that existing sampling would be a low-cost first step; we are not aware of a comparable program for pikas. These are suggestions for testing hypotheses, not a call to change control practice, and nothing in this study supports culling or poisoning either species.

### 4.12. Limitations

This study has several limitations. First, the pika model rests on a very small, spatially clustered occurrence sample (*n* = 17) that covers a narrow part of the regional climate (Section 4.10); the robustness analyses show that the direction of the projected loss is stable across occurrence filtering, regularization, binary thresholds and two climate models, but that it depends on the response to bio8 and on extrapolation beyond the sampled climate, so the magnitude of the pika’s projected collapse should be treated as provisional pending a larger, dedicated pika survey that includes records from warmer sites. Second, no future land-cover projection was available for the SSP3-7.0 pathway, and holding land cover at its current-day value for this scenario likely biases its area estimates toward those of the current scenario relative to the other three SSPs; relatedly, the primary future bioclimatic layers are drawn from a single global circulation model (BCC-CSM2-MR); a comparison with MIROC6 (Section 3.10) gave the same qualitative result, but two models at different resolutions do not span the uncertainty among GCMs, which diverge considerably in projected precipitation for semi-arid Inner Mongolia, so the magnitude of projected declines should be understood as conditional on the climate models used pending a multi-GCM ensemble comparison. Holding non-climatic layers constant had little effect on the pika projections (Table 4), but it excludes possible future changes in vegetation, land use and grazing. Third, the path model uses a linear specification to test a relationship that is, by construction of the JCI formula, partly non-linear (a geometric mean); its near-zero total effect should not be read as evidence that climate change has no net influence on JCI but rather reflects the limits of a linear model applied to a threshold-governed system, and the standardized path coefficients should be interpreted as a description of relative association, not as causal mediation: the suitability → JCI paths follow largely from how the index is calculated, and the climate → pika paths are not statistically distinguishable from zero under spatially corrected estimation. Fourth, the joint co-occurrence index and the path model built upon it are geographic and statistical proxies constructed from habitat suitability alone, in the absence of georeferenced brucellosis cases, serology or livestock-outbreak data for this region; they capture where and how strongly both candidate hosts are environmentally likely to co-occur, not validated transmission probability or realized disease burden, which depends additionally on host density, contact rates with livestock, pathogen prevalence within each host population, and numerous factors outside the scope of a habitat-suitability model [42]. Environmental suitability alone does not establish that either species is infected with *Brucella* in the study area, and we are not aware of published serological or isolation data for either species in Inner Mongolia. The only mention of brucellosis for *S. dauricus* that we found is a statement in a plague-surveillance paper that the species can also transmit brucellosis, given without supporting data [73], and the only figure for pikas is a review’s summary of serological surveys that reports a rate of 0.78% for “pikas” without naming the species [74]. Evidence from related taxa is also sparse: Richardson’s ground squirrels (*Spermophilus richardsonii*) orally inoculated with attenuated or virulent *Brucella abortus* still yielded the bacterium from tissues 14–18 weeks later, without pathology in non-pregnant animals [75], and 7.0% of Himalayan marmots (*Marmota himalayana*) sampled in Qinghai (80/1146) were seropositive, with a *B. abortus* biovar 1 isolate recovered from one marmot [76]. These studies show that related burrowing rodents can be exposed to or carry *Brucella*, but they do not show that *S. dauricus* or *O. dauurica* is infected, let alone that either maintains infection or transmits it to livestock; the designation of both species as candidate hosts therefore rests on their ecology and on evidence from related taxa, and remains a hypothesis. Likewise, a reduction in suitable habitat does not necessarily imply a proportional reduction in brucellosis incidence or spillover, which depend on livestock husbandry, vaccination, animal movement and human exposure, as well as on wildlife. In particular, the JCI says nothing about the spatial distribution of livestock themselves: brucellosis transmission risk to livestock depends on where grazing actually occurs, and Inner Mongolia’s grazing intensity is highly spatially heterogeneous and increasingly well mapped at fine resolution [77,78], so a high host co-occurrence zone that falls outside grazed land would carry limited practical epidemiological relevance; incorporating a livestock density or grazing-intensity layer as an explicit weighting term is a natural extension once such spatial data are available. We present this framework as an exploratory ecological analysis for generating hypotheses, not as a surveillance prioritization tool or as a calibrated risk or causal estimate, and recommend that it be validated against field seroprevalence data as such data become available. Fifth, consistent with the general concern that spatially structured raster data can inflate apparent sample size [49,79], both species’ occurrence records and background samples were restricted to Inner Mongolia, whereas their true accessible ranges extend into Mongolia and neighboring provinces; the M-area sensitivity check addresses this concern and finds the central conclusion robust to it, though the M-area sensitivity check itself relied on nearest-value extrapolation for static covariate layers in the buffer zone and coarser-resolution future climate data, so it should be read as directional confirmation rather than a full replacement for the primary analysis. Finally, both models were built from presence-only data with a shared background extent and, as with all correlative SDMs, do not capture biotic interactions, dispersal limitation, or evolutionary adaptation that could buffer against the projected declines [80,81].

## 5. Conclusions

Climate-driven habitat models for two candidate sylvatic brucellosis hosts in Inner Mongolia project sharply divergent futures. Under every future scenario examined, *Spermophilus dauricus* (a grassland generalist) undergoes a moderate, comparatively climate-trackable habitat decline, while *Ochotona dauurica* (a thermally constrained specialist) is projected to lose nearly all of its suitable habitat once a threshold in summer temperature is crossed, a result that is robust to occurrence filtering, regularization, binary thresholds and a second climate model but depends on the temperature response and on extrapolation beyond the climate range of the 17 records. A path analysis linking climate anomaly, host suitability change and JCI change describes an asymmetric rather than a parallel decline: warming predicts the squirrel’s habitat trajectory more linearly, yet the pika’s suitability change has nearly double the standardized association with JCI change; this association follows in part from how the index is constructed, and the climate effect on pika suitability is not statistically distinguishable from zero under spatially corrected estimation. An exploratory next-generation-matrix calculation, whose parameters are uncalibrated, shows the same direction of change under most parameter combinations but is sensitive to its assumptions (Table 2) and should not be read as a validated epidemiological prediction or as an estimate of epidemic potential.

Two practical implications follow directly from this asymmetry. First, for ecological monitoring, pika distribution shifts are the more informative indicator of change in the area where the two candidate hosts overlap as the region warms; whether pikas or ground squirrels matter more for *Brucella* maintenance or spillover is unknown and has to be established with serological, abundance and contact-rate data before surveillance resources are allocated. Second, the JCI and the exploratory transmission-threshold index developed here remain geographic and statistical proxies, not validated epidemiological predictions or measures of disease burden, until they are checked against field seroprevalence or case data, a validation step we recommend as the natural next stage of this work that would also help resolve the cross-species transmission efficiency this study can only bound rather than estimate. Pending that validation, this reproducible, transparent modeling framework (explicit variable screening, cross-validated model tuning, an openly documented JCI formula, a formal path model, and a next-generation-matrix transmission-dynamics extension) offers a template applicable beyond this system to any pastoral landscape where climate change is expected to reshape the overlap between multiple candidate wildlife hosts and livestock. In a One Health framing, this study supplies only the wildlife-distribution layer; linking it to livestock and human brucellosis requires coordinated serological and molecular sampling of both hosts alongside livestock and human case data (Section 4.11).

## Figures and Tables

**Figure 1:**
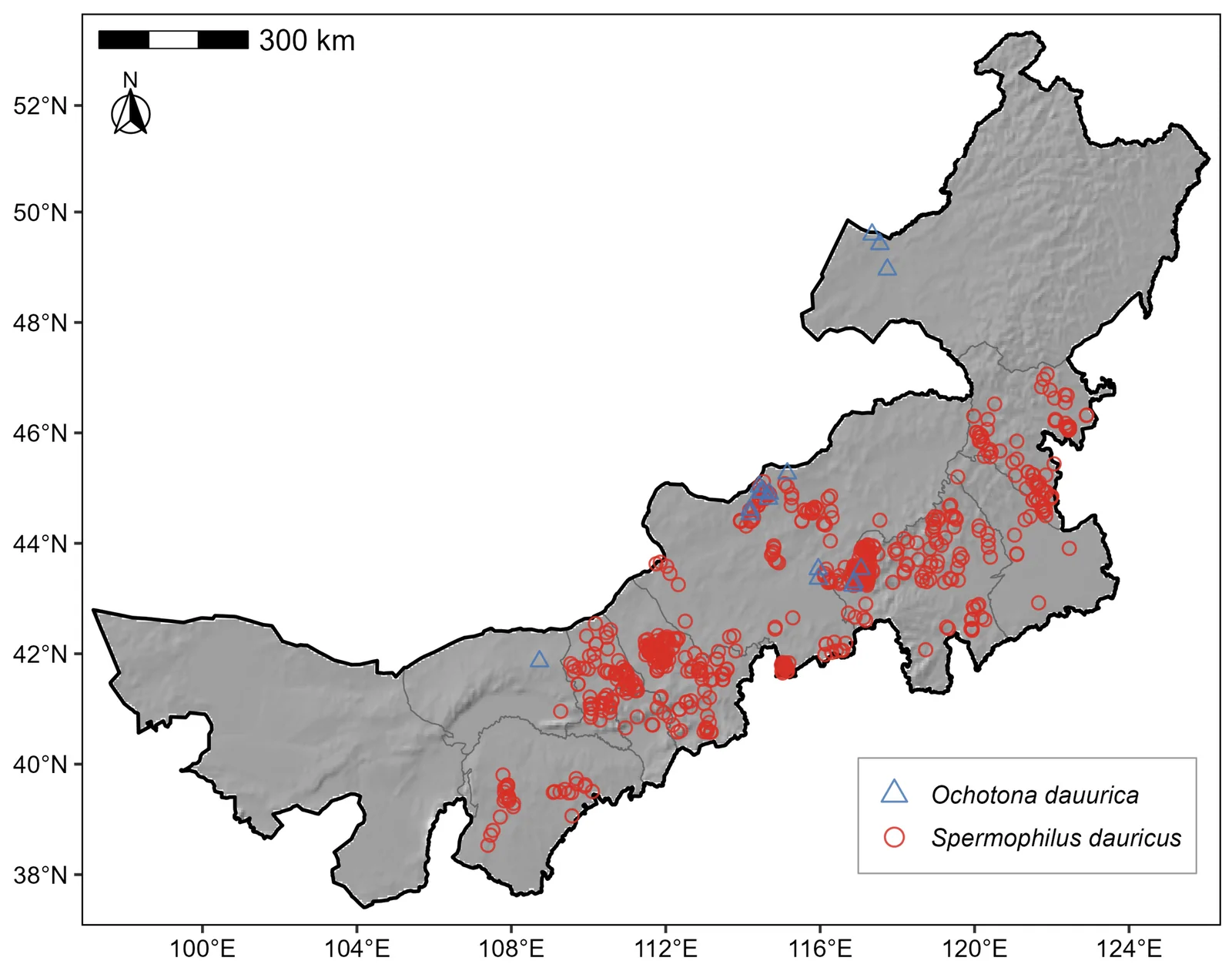
Study area (Inner Mongolia Autonomous Region, China) with prefecture-level (grey) and provincial (black) administrative boundaries, and occurrence records used for niche modeling: *Spermophilus dauricus* (red circles, *n* = 620) and *Ochotona dauurica* (blue triangles, *n* = 17).

**Figure 2:**
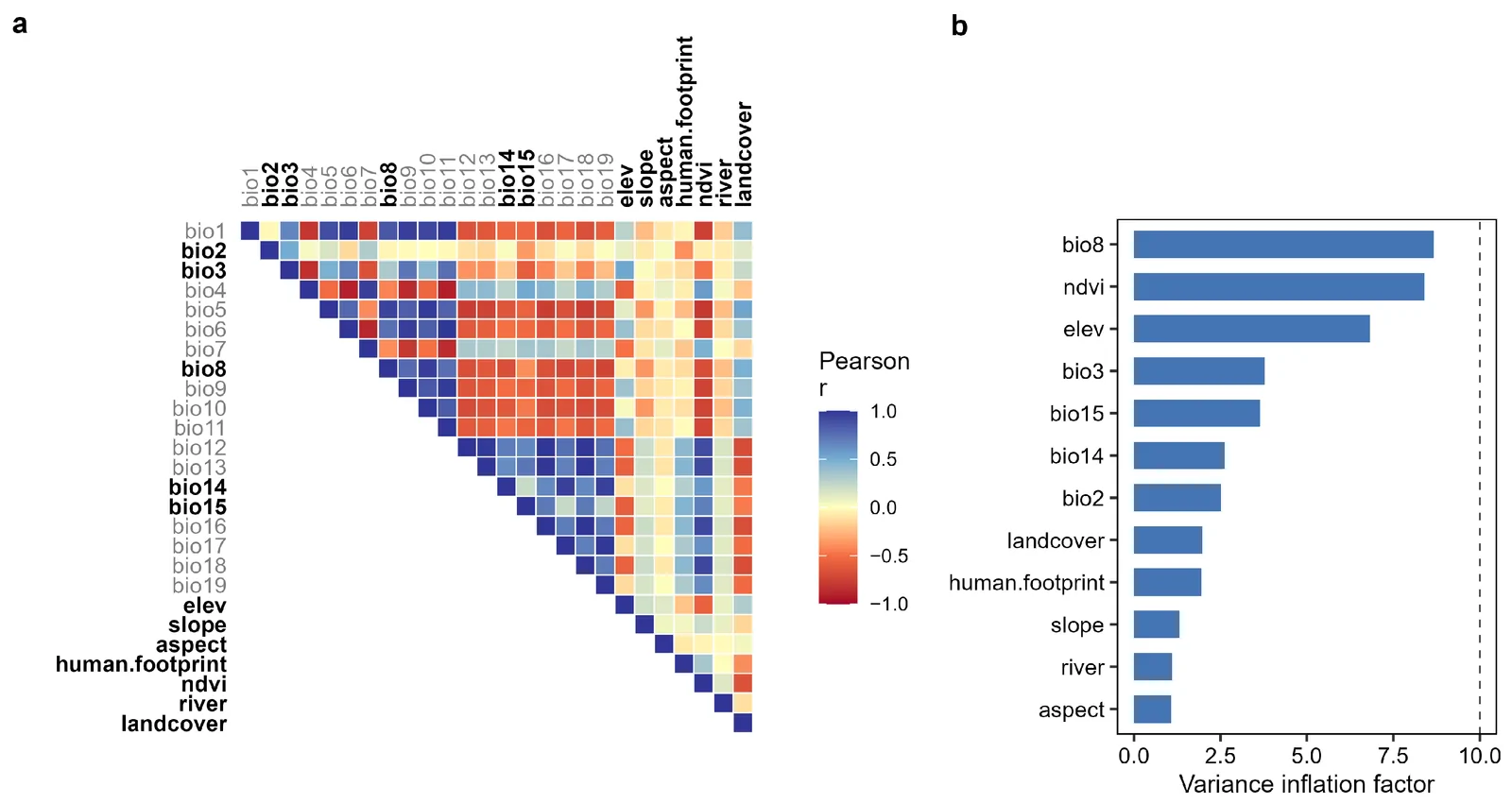
Environmental variable screening diagnostics (shared across both species, computed from the same background sample). (**a**) Pairwise Pearson correlation among the 26 candidate variables (bold labels: variables retained in the final 12-variable consensus set); (**b**) variance inflation factor for the 12 retained variables (dashed line: threshold = 10).

**Figure 3:**
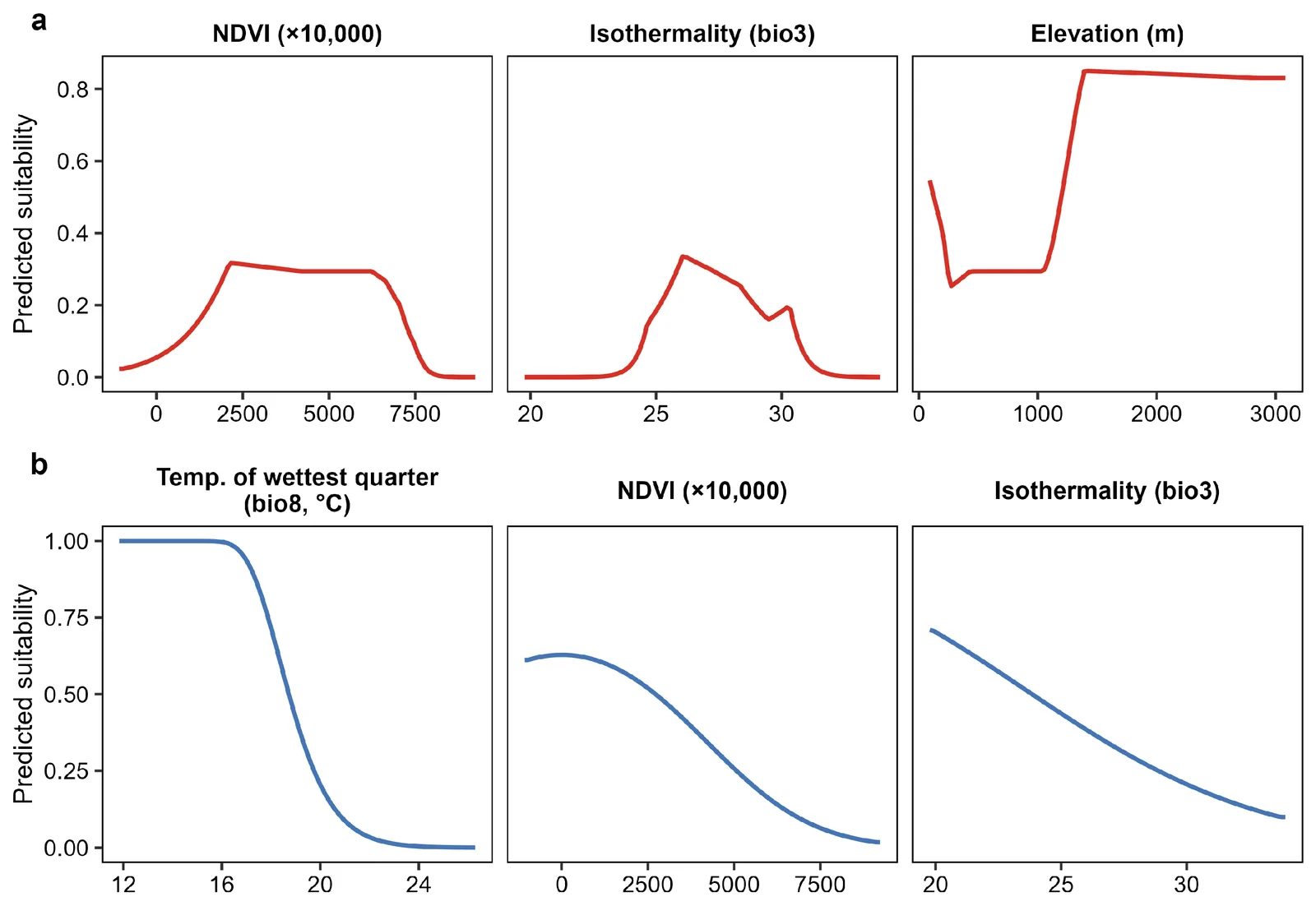
MaxEnt response curves for the top three predictors (by permutation importance on the final model) for (**a**) *Spermophilus dauricus* and (**b**) *Ochotona dauurica*. NDVI is shown in the scaled units of the source raster (NDVI × 10,000).

**Figure 4:**
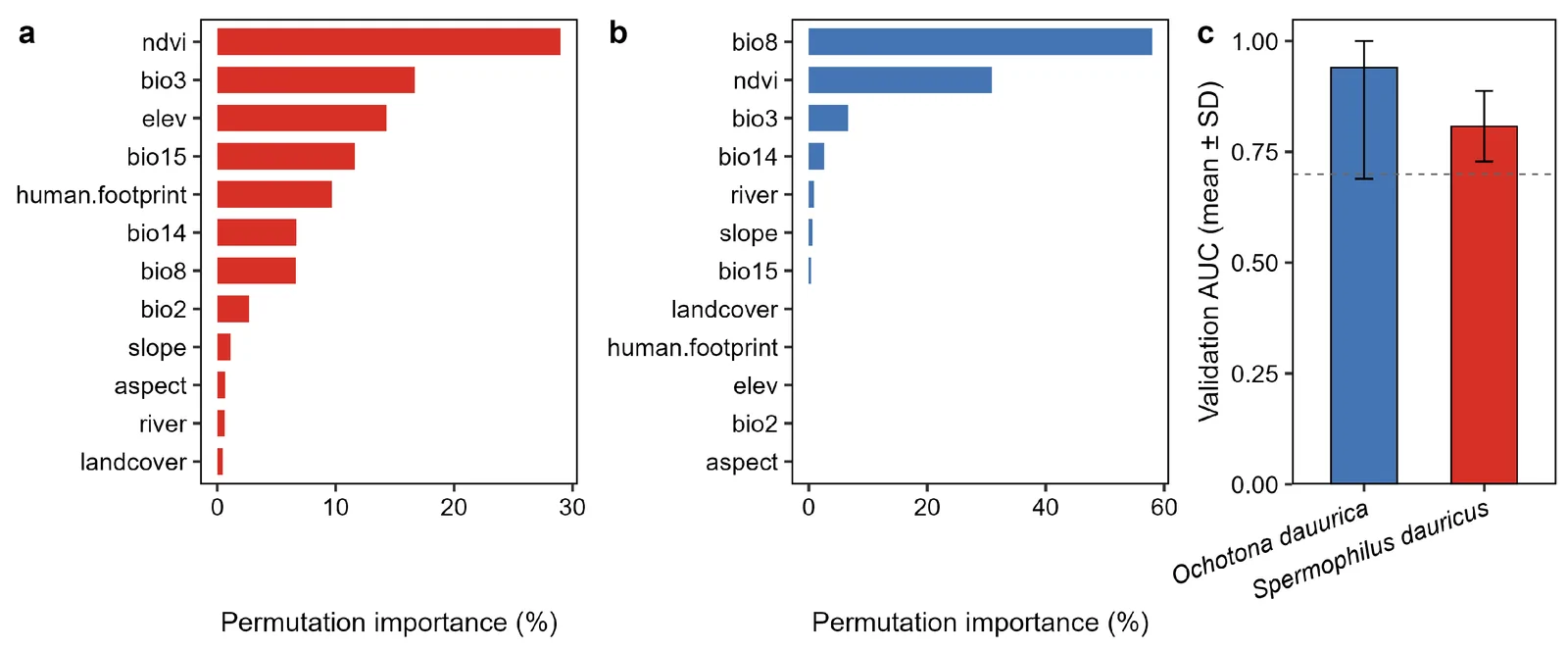
ENMeval-optimized MaxEnt model performance and permutation variable importance for (**a**) *Spermophilus dauricus* and (**b**) *Ochotona dauurica*; (**c**) validation AUC (mean ± SD across cross-validation folds) for both species, with the dashed line marking AUC = 0.7, a commonly used threshold for acceptable discrimination.

**Figure 5:**
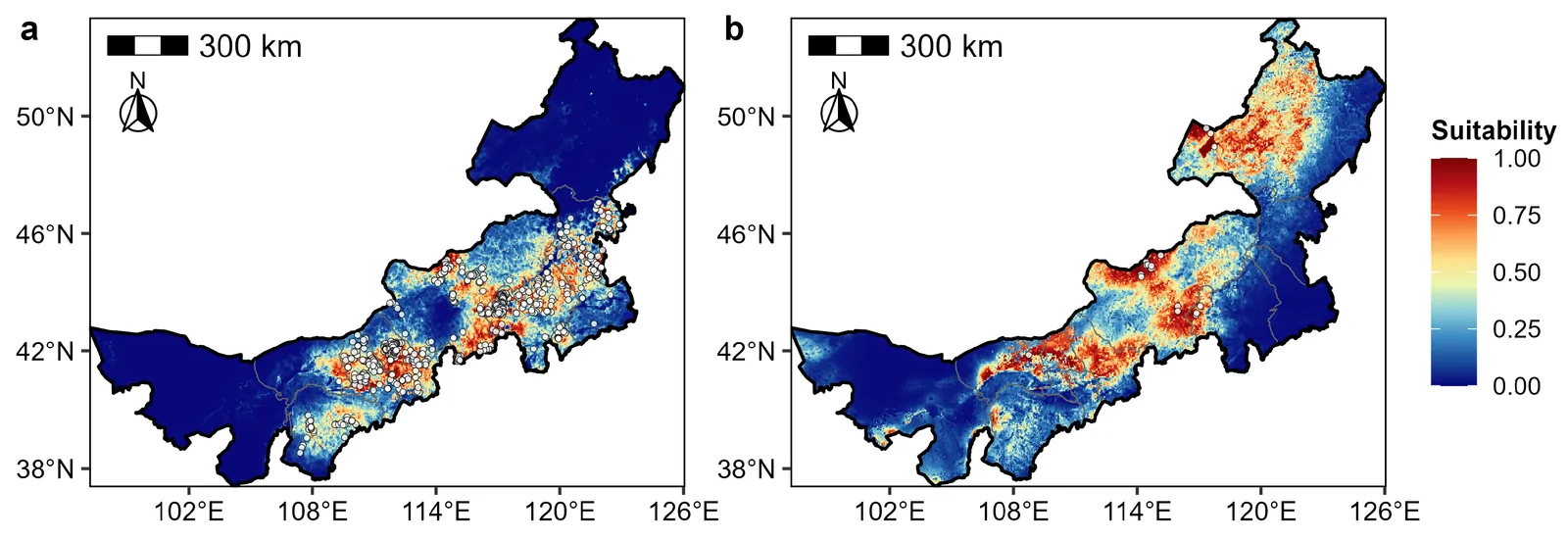
Current predicted habitat suitability from the ENMeval-optimized MaxEnt models for (**a**) *Spermophilus dauricus* and (**b**) *Ochotona dauurica*, with occurrence records overlaid (white circles).

**Figure 6:**
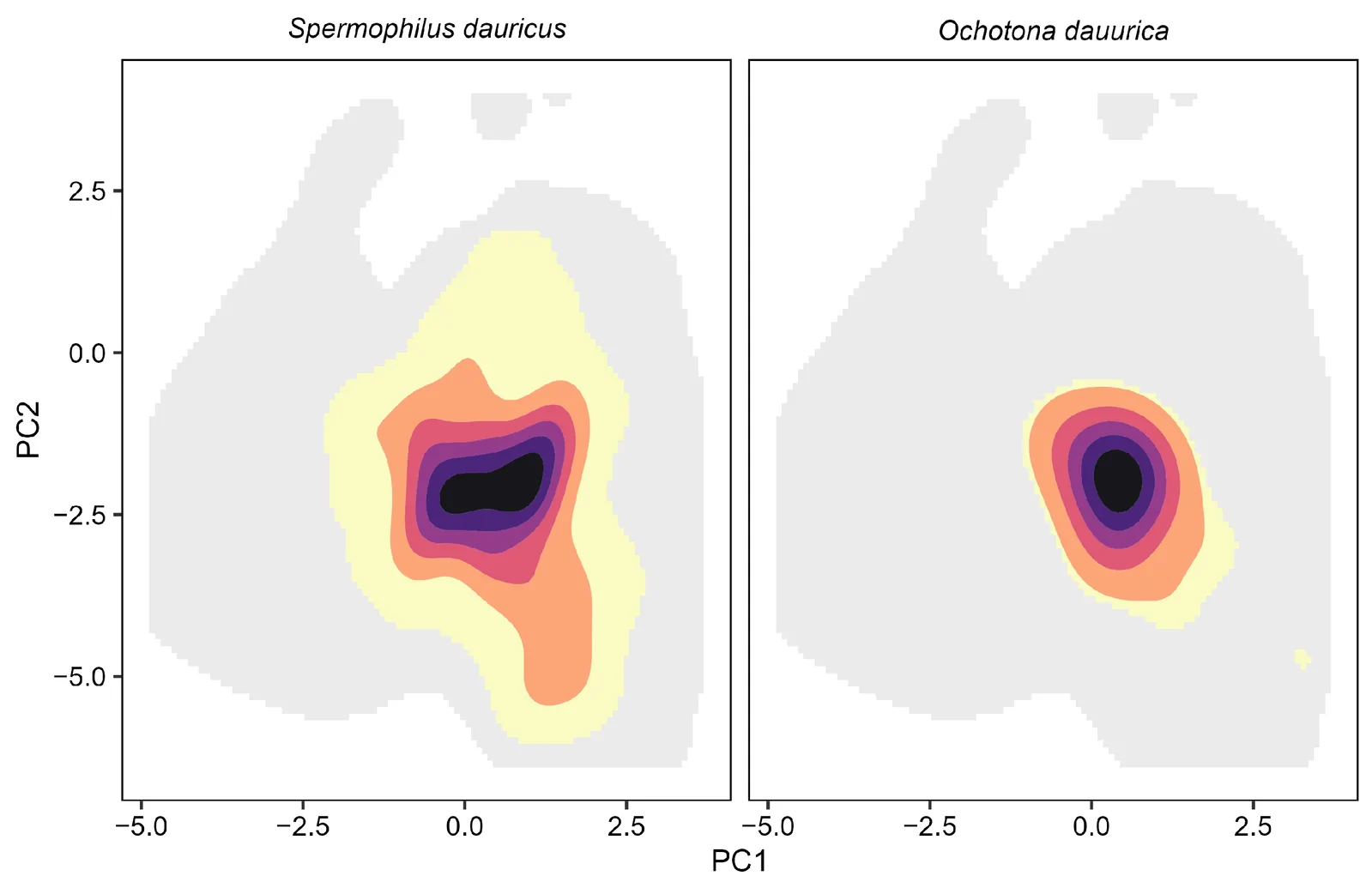
Interspecific environmental niche overlap between *Spermophilus dauricus* (**left**) and *Ochotona dauurica* (**right**) in a shared PCA-environment space built from 10 continuous predictors. Grey: environment available in the study region; filled contours: occurrence density (from pale yellow, lowest, to black, highest) in six equal bins from the kernel-smoothed density surface.

**Figure 7:**
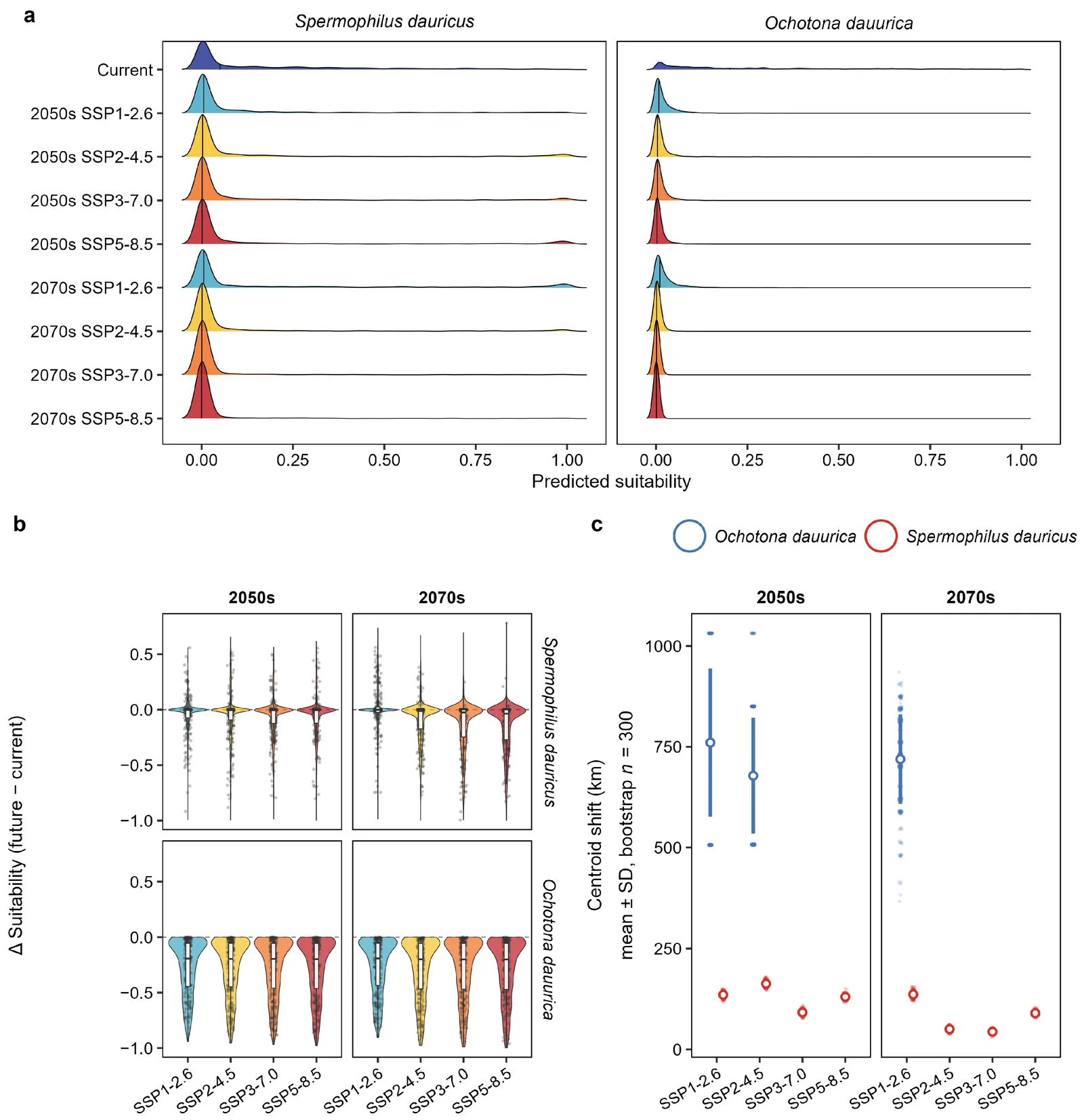
Projected range change under eight future climate scenarios (2050s/2070s × SSP1-2.6/2-4.5/3-7.0/5-8.5), shown as full distributions rather than single summary percentages. (**a**) Ridgeline plot of the landscape suitability-score distribution for current climate and each future scenario, for *Spermophilus dauricus* and *Ochotona dauurica* (vertical line: median); (**b**) per-cell suitability change (future–current) distribution, faceted by species and period; (**c**) bootstrap distribution (*n* = 300 resamples per scenario) of the suitability-weighted centroid-shift distance for both species, mean ± SD; pika estimates are shown only for the three scenarios retaining more than one suitable grid cell since a single-cell suitable area gives a degenerate, zero-variance bootstrap. In (**a**,**b**), colors indicate the climate scenario (dark blue, current climate; light blue, SSP1-2.6; yellow, SSP2-4.5; orange, SSP3-7.0; red, SSP5-8.5). Δ denotes the change in suitability between future and current climate; in (**b**), boxes show the median and interquartile range and dots a random subsample of grid cells; in (**c**), open circles with bars show the mean ± SD and faint dots individual bootstrap values (blue, *Ochotona dauurica*; red, *Spermophilus dauricus*).

**Figure 8:**
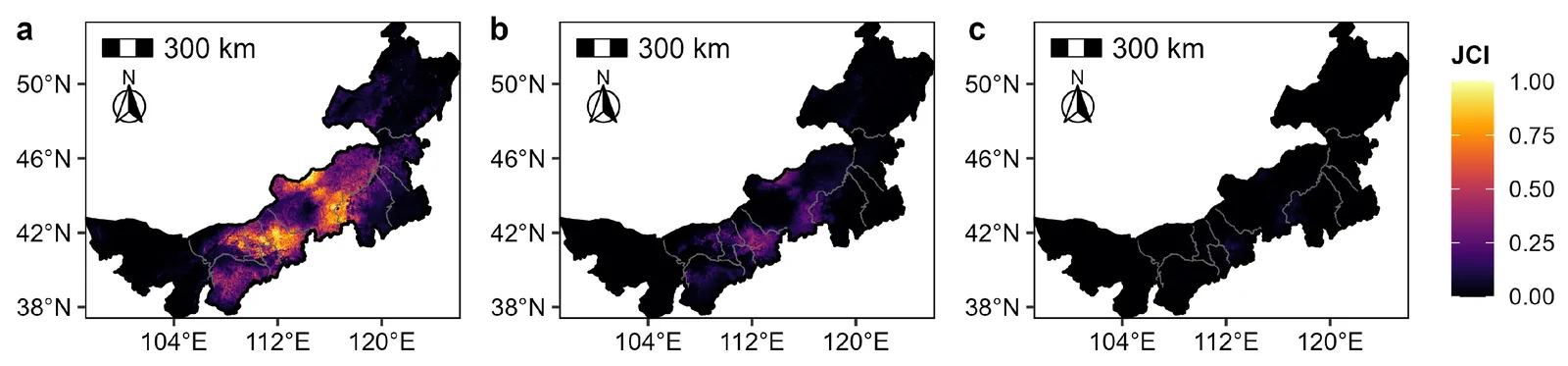
Joint host co-occurrence index (JCI) under (**a**) current climate, (**b**) the mildest future scenario examined (2050s SSP1-2.6), and (**c**) the most severe (2070s SSP5-8.5).

**Figure 9:**
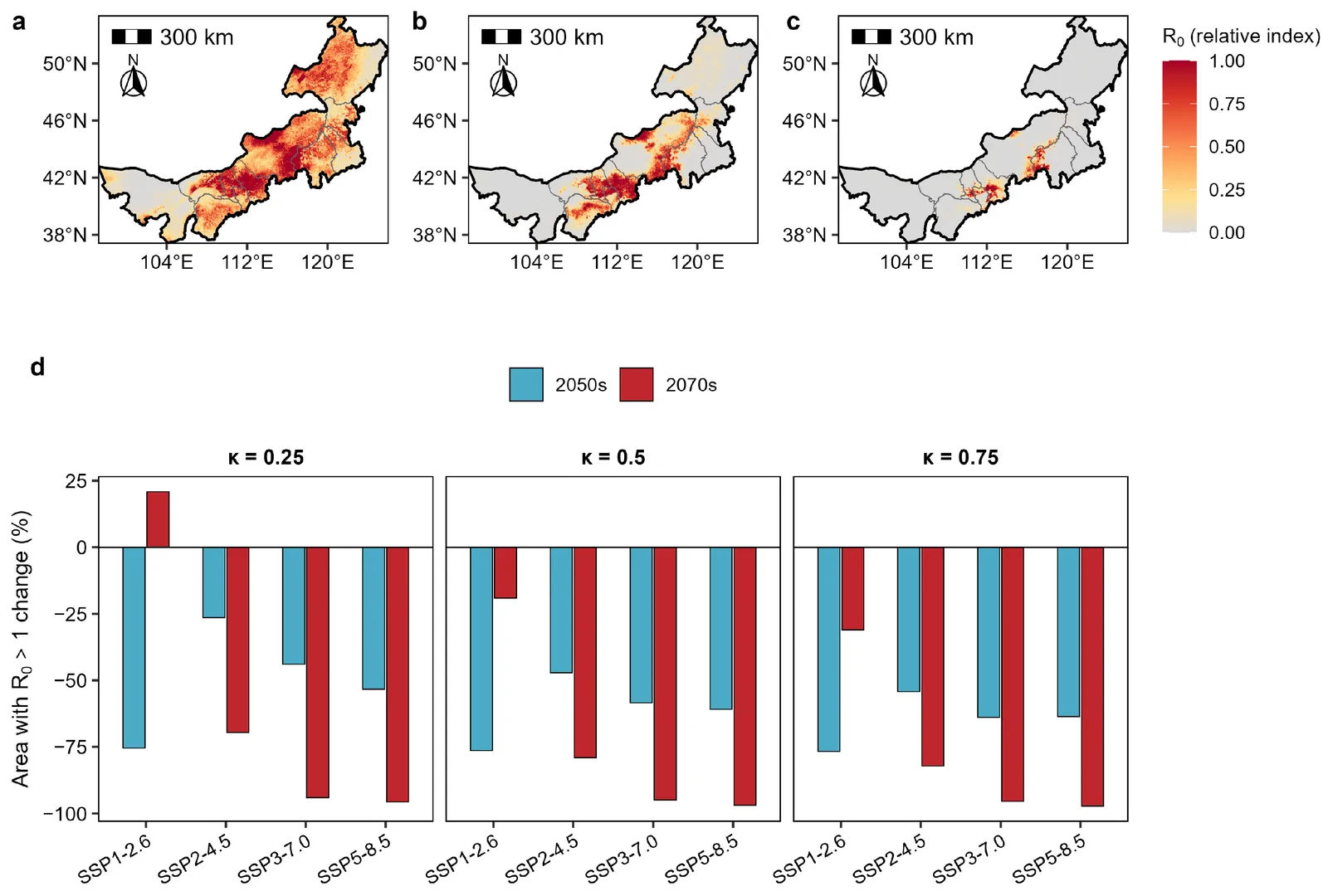
Exploratory transmission-threshold analysis (relative next-generation-matrix *R*_0_ index; not a validated epidemiological prediction). (**a**–**c**) *R*_0_ maps at *κ* = 0.5 for current climate, the mildest future scenario (2050s SSP1-2.6), and the most severe (2070s SSP5-8.5). (**d**) Percent change in the area with *R*_0_ > 1 relative to current, across all eight future scenarios and the three *κ* (cross-/within-species transmission ratio) sensitivity values. No additional flooring or truncation was applied beyond the next-generation-matrix formula itself; near-zero suitability necessarily yields near-zero *R*_0_ through the formula’s own structure.

**Figure 10:**
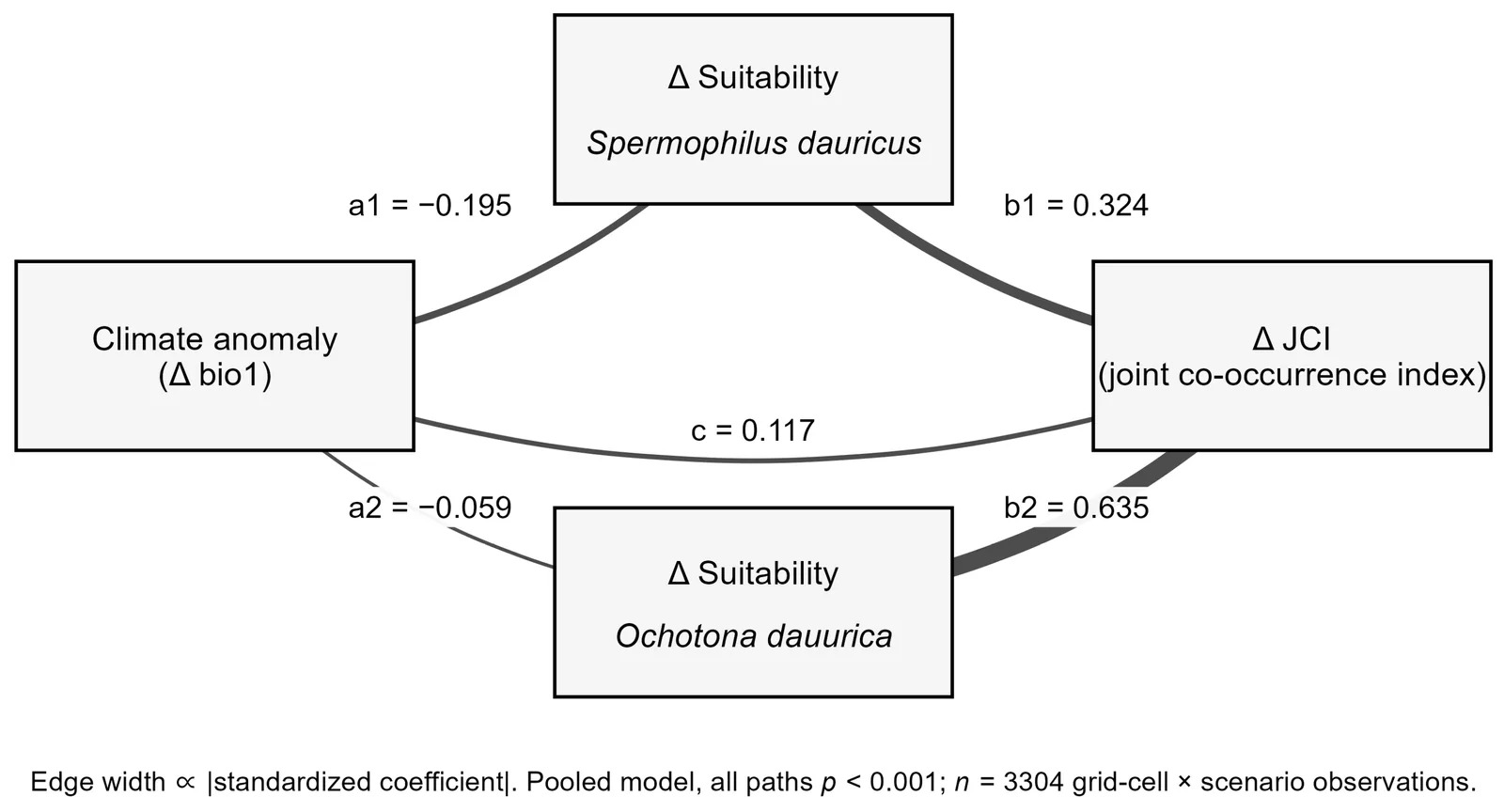
Path diagram of climate anomaly, suitability change and JCI change. Standardized path coefficients from the lavaan path model linking climate anomaly (Δbio1) to change in each species’ suitability, and each species’ suitability change to change in joint host co-occurrence index (*n* = 3304 pooled grid-cell × scenario observations; edge width proportional to |standardized coefficient|; all paths *p* < 0.001). Coefficients are from a linear approximation of a system with at least one strongly non-linear, threshold-governed component; spatial-autocorrelation diagnostics and robustness checks are given in the text. Δ denotes change from current to future climate; a1 and a2 are the climate → suitability paths, b1 and b2 the suitability → JCI paths, and c the direct climate → JCI path.

**Figure 11:**
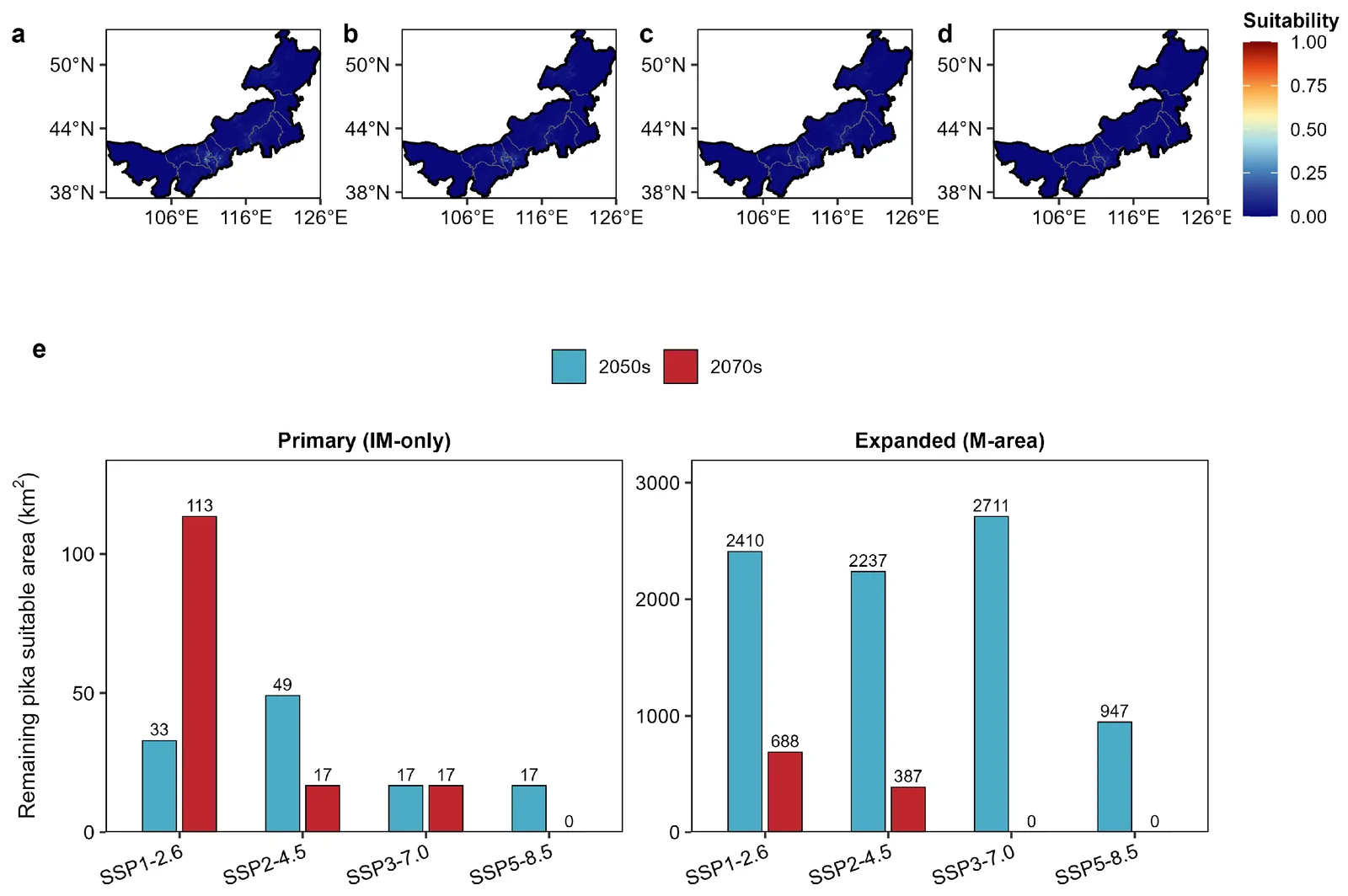
Background-extent (M-area) sensitivity check extended to future climate. (**a**–**d**) Expanded-background pika suitability within Inner Mongolia for the four SSP pathways (2070s, real CMIP6 MIROC6 data, 10 arc-minute resolution). (**e**) Remaining pika suitable area (km^2^) by SSP pathway and period (2050s/2070s), for the primary (Inner-Mongolia-only) and expanded-background (M-area) models, each on its own y-axis; despite the expanded model’s larger current-climate baseline (213,485 km^2^ versus 136,458 km^2^ under the primary model), both models show near-total loss (≥98.7%) of currently suitable area by the 2070s under every scenario, falling to zero mapped suitable cells under two of the four 2070s scenarios for the expanded model and one for the primary model.

**Figure 12:**
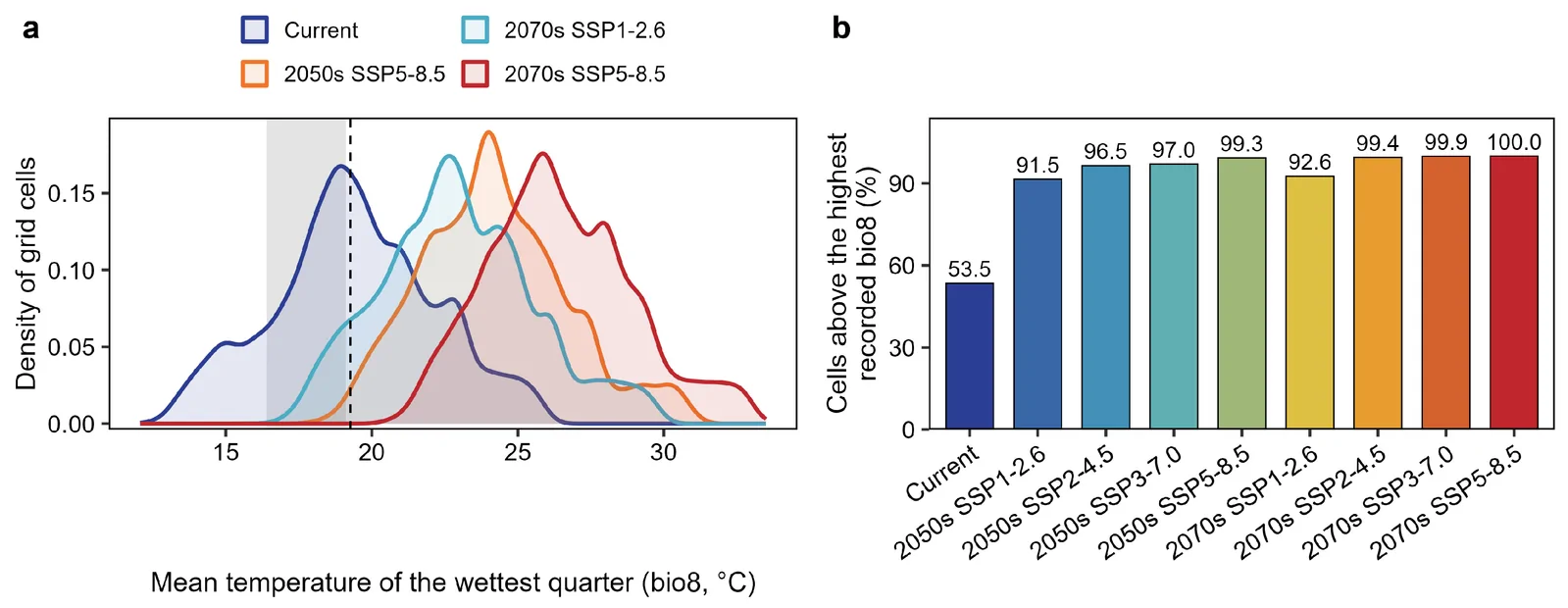
Extrapolation of the pika’s temperature response. (**a**) Density of mean temperature of the wettest quarter (bio8) across Inner Mongolia grid cells under current climate and three future scenarios (BCC-CSM2-MR); the grey band is the range sampled by the 17 pika records (16.40–19.12 °C) and the dashed line is the bio8 value at which the primary model’s predicted suitability crosses 0.5 (19.27 °C). (**b**) Percentage of Inner Mongolia grid cells in which bio8 exceeds the highest value at a pika record, under current climate and each future scenario.

**Table 1:** Suitable area, area change relative to current, and host co-occurrence overlap area under current climate and eight future scenarios.

Scenario	Squirrel Area (km^2^)	Squirrel Δ (%)	Pika Area (km^2^)	Pika Δ (%)	Overlap Area (km^2^)	Overlap Δ (%)
Current	301,371	—	136,458	—	66,737	—
2050s SSP1-2.6	201,471	−33.1	33	−99.98	16	−99.98
2050s SSP2-4.5	189,027	−37.3	49	−99.96	32	−99.95
2050s SSP3-7.0 *	160,832	−46.6	17	−99.99	0	−100
2050s SSP5-8.5	156,705	−48.0	17	−99.99	0	−100
2070s SSP1-2.6	272,564	−9.6	113	−99.92	0	−100
2070s SSP2-4.5	116,871	−61.2	17	−99.99	0	−100
2070s SSP3-7.0 *	55,772	−81.5	17	−99.99	0	−100
2070s SSP5-8.5	47,225	−84.3	0	−100	0	−100

Note: SSP3-7.0 area estimates were derived using current-day land cover; Δ = percent change relative to current climate. * Rows marked with an asterisk should be interpreted with this land-cover caveat in mind when compared to the other three SSPs.

**Table 2:** Sensitivity of the area with *R*_0_ > 1 (*κ* = 0.5) to the parameterization of the next-generation matrix. Change is the percent change in area relative to current climate, given as the range across the eight future scenarios.

Assumption	Area with *R*_0_ > 1, Current Climate (km^2^)	Change Across Eight Scenarios (%)	Scenarios with an Increase
Baseline (γ = 1, ρ = 1, s = 1)	64,033	−96.9 to −19.0	0
γ = 0.5	158,413	−95.9 to −34.2	0
γ = 2	23,863	−99.5 to −10.8	0
ρ = 0.5	17,637	−91.1 to +138.6	4
ρ = 2	286,828	−99.1 to −77.2	0
s = 0.5	0	–	–
s = 2	393,948	−92.3 to −47.0	0

γ, exponent relating suitability to relative host density; ρ, ratio of the pika’s to the squirrel’s within-species transmission coefficient; s, common scale of the within-species coefficients.

**Table 3:** Standardized path coefficients and indirect effects with 95% confidence intervals for the pooled lavaan model (*n* = 3304) and the spatial generalized least-squares (GLS) models fitted to z-scored variables (*n* = 536; intervals for indirect effects from Monte Carlo propagation).

Path	Pooled Lavaan, β [95% CI]	Spatial GLS, β [95% CI]
a_1_: climate anomaly → squirrel suitability	−0.195 [−0.227, −0.162]	−0.181 [−0.304, −0.058]
a_2_: climate anomaly → pika suitability	−0.059 [−0.093, −0.025]	−0.061 [−0.305, 0.184]
b_1_: squirrel suitability → JCI	0.324 [0.300, 0.349]	0.256 [0.206, 0.307]
b_2_: pika suitability → JCI	0.635 [0.615, 0.654]	0.601 [0.545, 0.657]
c: climate anomaly → JCI (direct)	0.117 [0.092, 0.141]	0.132 [−0.040, 0.305]
Indirect effect via squirrel (a_1_b_1_)	−0.063 [−0.075, −0.051]	−0.046 [−0.081, −0.014]
Indirect effect via pika (a_2_b_2_)	−0.038 [−0.059, −0.016]	−0.037 [−0.185, 0.109]
Total indirect effect	−0.101 [−0.125, −0.076]	−0.083 [−0.235, 0.066]
Total effect	0.016 [−0.018, 0.050]	–

JCI, joint host co-occurrence index. The total effect was not computed for the GLS models.

**Table 4:** Robustness of the projected loss of suitable habitat for *Ochotona dauurica* to model specification, occurrence data, binary threshold, non-climatic layers and climate model. Decline is the percent loss of suitable area relative to current climate, given as the range across the eight future scenarios; RM, regularization multiplier; LQ, linear + quadratic features; L, linear features; RM and settings as in the primary model unless stated.

Variant	Records (n)	Current Suitable Area (km^2^)	Decline Across Eight Scenarios (%)	Scenarios with ≥99% Decline
Primary model (LQ, RM = 4; stored)	17	136,458	99.9–100	8
LQ, RM = 2	17	156,058	99.7–100	8
LQ, RM = 1	17	99,330	99.3–100	8
L features only, RM = 2	17	166,197	72.9–99.5	1
LQ, RM = 4, bio8 excluded	17	174,342	+54.9 to +151.7 (increase)	0
LQ, RM = 4, climate predictors only	17	186,339	98.9–100	7
Thinned to >10 km	14	137,795	99.9–100	8
Thinned to >20 km	11	140,280	99.9–100	8
Thinned to >40 km	8	141,572	99.4–100	8
50 random subsamples (median)	12	121,774	99.9–100	8
Threshold: minimum training presence (=MaxSSS)	17	196,597	99.9–100	8
Threshold: 20th percentile of presences	17	71,993	100	8
Threshold-free: landscape-mean suitability	17	–	91.8–99.6	2
Second climate model (MIROC6), primary model	17	136,458	100	8
Non-climatic layers held at current values	17	136,458	99.9–100	8

For the random subsamples, values are medians over 50 replicates. For the threshold-free metric, the decline refers to landscape-mean suitability.

## Data Availability

The pika occurrence data supporting this study are available from GBIF.org [30]. The ground squirrel field-survey dataset and derived environmental raster layers analyzed in this study are available from the corresponding author on reasonable request. The analysis code is available from the corresponding author on reasonable request.

## Abbreviations

The following abbreviations are used in this manuscript:

SDM: species distribution model; MaxEnt: maximum entropy; AUC: area under the receiver operating characteristic curve; AICc: corrected Akaike Information Criterion; VIF: variance inflation factor; NDVI: normalized difference vegetation index; GCM: global circulation model; CMIP6: Coupled Model Intercomparison Project Phase 6; SSP: Shared Socioeconomic Pathway; NGM: next-generation matrix; *R*_0_: basic reproduction number; GLS: generalized least squares; PCA: principal component analysis; GBIF: Global Biodiversity Information Facility; JCI: joint host co-occurrence index; M-area: background/accessible environmental extent used for model training.
